# Contingency Management for Patients Receiving Medication for Opioid Use Disorder: A Systematic Review and Meta-analysis

**DOI:** 10.1001/jamapsychiatry.2021.1969

**Published:** 2021-08-04

**Authors:** Hypatia A. Bolívar, Elias M. Klemperer, Sulamunn R. M. Coleman, Michael DeSarno, Joan M. Skelly, Stephen T. Higgins

**Affiliations:** 1 Vermont Center on Behavior and Health, University of Vermont, Burlington; 2 Department of Psychiatry, University of Vermont, Burlington; 3 Medical Biostatistics, University of Vermont, Burlington; 4 Department of Psychological Science, University of Vermont, Burlington

## Abstract

**Importance:**

Medication treatment for opioid use disorder (MOUD) is efficacious, but comorbid stimulant use and other behavioral health problems often undermine efficacy.

**Objective:**

To examine the association of contingency management, a behavioral intervention wherein patients receive material incentives contingent on objectively verified behavior change, with end-of-treatment outcomes for these comorbid behavioral problems.

**Data Sources:**

A systematic search of PubMed, Cochrane CENTRAL, Web of Science, and reference sections of articles from inception through May 5, 2020. The following search terms were used: *vouchers* OR *contingency management* OR *financial incentives.*

**Study Selection:**

Prospective experimental studies of monetary-based contingency management among participants receiving MOUD.

**Data Extraction and Synthesis:**

Following Preferred Reporting Items for Systematic Reviews and Meta-analyses (PRISMA) reporting guideline, 3 independent investigators extracted data from included studies for a random-effects meta-analysis.

**Main Outcomes and Measures:**

Primary outcome was the association of contingency management at end-of-treatment assessments with 6 clinical problems: stimulant use, polysubstance use, illicit opioid use, cigarette smoking, therapy attendance, and medication adherence. Random-effects meta-analysis models were used to compute weighted mean effect size estimates (Cohen *d*) and corresponding 95% CIs separately for each clinical problem and collapsing across the 3 categories assessing abstinence and the 2 assessing treatment adherence outcomes.

**Results:**

The search identified 1443 reports of which 74 reports involving 10 444 unique adult participants met inclusion criteria for narrative review and 60 for inclusion in meta-analyses. Contingency management was associated with end-of-treatment outcomes for all 6 problems examined separately, with mean effect sizes for 4 of 6 in the medium-large range (stimulants, Cohen *d* = 0.70 [95% CI, 0.49-0.92]; cigarette use, Cohen *d* = 0.78 [95% CI, 0.43-1.14]; illicit opioid use, Cohen *d* = 0.58 [95% CI, 0.30-0.86]; medication adherence, Cohen *d* = 0.75 [95% CI, 0.30-1.21]), and 2 in the small-medium range (polysubstance use, Cohen *d* = 0.46 [95% CI, 0.30-0.62]; therapy attendance, *d* = 0.43 [95% CI, 0.22-0.65]). Collapsing across abstinence and adherence categories, contingency management was associated with medium effect sizes for abstinence (Cohen *d* = 0.58; 95% CI, 0.47-0.69) and treatment adherence (Cohen *d* = 0.62; 95% CI, 0.40-0.84) compared with controls.

**Conclusions and Relevance:**

These results provide evidence supporting the use of contingency management in addressing key clinical problems among patients receiving MOUD, including the ongoing epidemic of comorbid psychomotor stimulant misuse. Policies facilitating integration of contingency management into community MOUD services are sorely needed.

## Key Points

**Question:**

Is contingency management associated with outcomes for treating comorbid substance use and treatment nonadherence among patients receiving medication for opioid use disorder?

**Findings:**

In this systematic review and meta-analysis that included 74 randomized clinical trials and 10 444 adults receiving medication for opioid use disorder, the efficacy of contingency management was associated with abstinence from 4 types of substance use (psychomotor stimulants, polysubstance use, illicit opioids, and cigarettes) and improved treatment attendance and medication adherence.

**Meaning:**

These results provide evidence supporting the use of contingency management for addressing common and serious clinical problems among patients receiving medication for opioid use disorder.

## Teaser

This systematic review and meta-analysis examines the efficacy of contingency management for treating comorbid behavioral problems in individuals receiving medication for opioid use disorder.

## Introduction

The opioid epidemic remains a US public health crisis, with more than 10 million people in the US 12 years and older (3.7% of the US population) reporting past-year opioid misuse.^1^ Ongoing opioid use in the US has resulted in a tragic death toll and substantial financial burden. For example, nearly 70% of US drug overdoses involve opioids,^2^ which contributed to an overall decrease in mean life expectancy in the US.^3^ The estimated annual economic cost of opioid use disorder (OUD) in the US exceeds $786 billion.^4^

OUD is often accompanied by other substance use and barriers to treatment adherence. Past-month nonopioid drug use was reported by 97% of nearly 16 000 patients entering OUD treatment between 2011 and 2018 in the US.^5^ Medication for OUD (MOUD) is highly effective in reducing illicit opioid use and associated adverse outcomes,^6^ but surging psychomotor stimulant use^7^ can undermine efficacy contributing to premature treatment termination and return to illicit drug use.^8^ Recent increases in psychomotor stimulant use among people with OUD is highly concerning, with overdose deaths from psychomotor stimulant use more than doubling between 2011 and 2017.^9^ There is concern that this surge in psychomotor stimulant use among those with OUD has potential to undermine the considerable progress made in curtailing the opioid crisis through MOUD.

Promising research has emerged on several types of medications or medication combinations for stimulant use disorders, but effects are inconsistent and effect sizes are generally small.^10,11^ Thus, treatment of psychomotor stimulant use requires psychosocial interventions. A 2018 meta-analysis of 50 randomized clinical trials found that contingency management was the only intervention that was associated with a significant reduction in stimulant use.^12^ Prior reviews noted that contingency management effectively reduced nonprescribed drug use among populations with OUD^13,14^ but are now dated or failed to address treatment adherence.

The overarching aim of this systematic review and meta-analysis is to provide a timely and comprehensive review of contingency management’s efficacy in addressing the public health crisis of psychomotor stimulant use and other common clinical challenges among patients receiving MOUD. Such evidence could be critically important to improving MOUD outcomes and advancing the Biden/Harris administration’s priority of increasing contingency management accessibility.^15^

## Methods

This review was conducted following Preferred Reporting Items for Systematic Reviews and Meta-analyses (PRISMA) reporting guideline. The protocol was submitted to Prospective Register of Systematic Reviews in November 2019 after piloting study selection process but prior to formal screening of search results.

### Search Strategy

**Figure 1.**
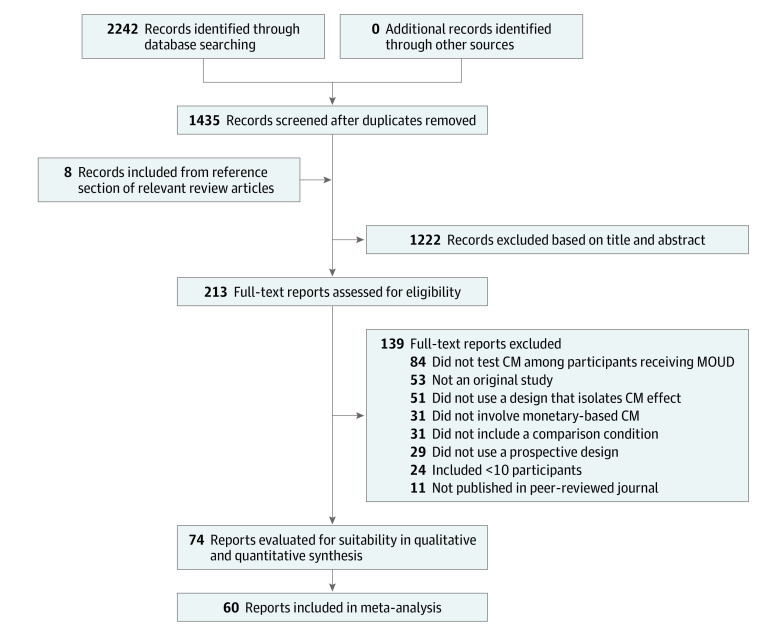
PRISMA Diagram of Included and Excluded Reports CM indicates contingency management; MOUD, medication for opioid use disorder.

We searched PubMed, Web of Science, and Cochrane Controlled Register of Trials (CENTRAL) databases to identify studies examining contingency management with patients receiving MOUD published from inception to May 6, 2020. We searched PubMed and Cochrane CENTRAL using the terms *vouchers* OR *contingency management* OR *financial incentives* [all fields] AND (*substance-related disorders* [MeSH]). We searched Web of Science using the terms *vouchers* OR *contingency management* OR *financial incentives* [all fields] AND (*substance abuse* [research area]). Additionally, we hand-searched reference sections of relevant reports. Reports had to be published in English. Our search identified 1435 reports. We identified 8 additional reports by hand search for a total of 1443 reports for initial screening (Figure 1). No observational studies were included.

### Study Selection

At least 2 authors (H.A.B., E.M.K., and S.R.M.C.) screened abstracts and titles of these 1443 reports to determine eligibility for full-text review. Empirical studies of contingency management for substance use or treatment adherence in a population receiving MOUD were selected for full-text review. A total of 213 reports were advanced for full-text review to determine inclusion using the following criteria: (1) appears in a peer-reviewed journal, (2) reports results from an original study, (3) tests a monetary-based contingency management intervention, (4) uses a prospective between- or within-participant experimental design, (5) includes a no-incentives comparison condition, (6) reports findings from 10 or more participants, (7) uses a research design allowing attribution of treatment effects to contingency management, and (8) reports findings in which all participants received MOUD or a subanalysis in which data were exclusively from participants who received MOUD. Based on these criteria, 139 reports were excluded (eTable 7 in the Supplement), leaving 74 that met full inclusion criteria (eTables 1-5 in the Supplement).

### Data Extraction

Two or more authors (H.A.B., E.M.K., and S.R.M.C.) independently read the full text of the 74 reports that met inclusion criteria to determine the clinical problem being treated and extracted the data summarized in eTables 1 to 5 in the Supplement. We calculated maximum daily earnings by dividing maximum total earnings possible by number of days contingency management was provided. Discrepancies in data extraction were resolved through discussion until consensus was reached. If outcomes of interest were only reported graphically, we obtained data using a tool for extracting numerical data estimates from figures.^16^

Outcomes at the end of treatment were the primary outcome; treatment effects at longest follow-up after contingency management was discontinued was a secondary outcome. Of 74 reports included, 71 (96%) reported end-of-treatment outcomes and 3 (4%) reported only follow-up outcomes.^17,18,19^ For studies targeting abstinence, longest duration of continuous abstinence was prioritized over other abstinence outcomes (eg, percent drug-negative urine samples) because it most closely approximates quitting use and is a robust predictor of longer-term abstinence.^20^ This review examined 6 common problems among patients receiving MOUD: (1) psychomotor stimulant use, (2) polysubstance use, (3) illicit opioid use, (4) cigarette smoking, (5) therapy attendance, and (6) medication adherence.

### Quality Assessment

Using the Effective Public Health Practice Project tool, 2 or more reviewers (H.A.B., E.M.K., and S.R.M.C.) independently rated each study on selection bias, study design, confounders, blinding, data collection methods, withdrawals and dropouts, intervention integrity, and appropriateness of analysis.^21^ Discrepancies were resolved via discussion until consensus was reached. We determined that blinding was impractical for the current review given that contingency management is a behavioral intervention. Thus, we report quality assessment scores (1 = strong, 2 = moderate, and 3 = weak) in eTables 1 to 5 in the Supplement for each included study excluding the blinding rating; scores including the blinding rating are reported in eTable 6 in the Supplement.

### Statistical Analysis

Cohen *d* was used to measure effect size.^22^ Positive values of Cohen *d* correspond with a superior outcome for contingency management compared with control. Whenever possible, effect sizes were computed based on the reported test statistic. In cases where this was unavailable, effect sizes were computed using descriptive statistics. For studies in which multiple contingency management conditions were compared with control, individual effect sizes were computed for each contingency management condition compared with control. In some cases, where deemed appropriate, effect sizes were computed for combined contingency management conditions vs control or vs combined control conditions. For studies with effect sizes for multiple comparisons or multiple outcomes, mean effect sizes were calculated across comparisons and outcomes to generate an overall study effect size. Random-effects meta-analysis models were used to compute weighted mean effect size estimates and corresponding 95% CIs for each selected subset of studies. These random-effects models weight each study’s effect in inverse proportion to its variance. We tested study quality rating, duration of contingency management, and maximum daily earnings as potential moderators given their clinical relevance^23^ and potential to account for heterogeneity across studies. We generated a funnel plot and examined sample size as a moderator of effect size across all included studies to assess for possible publication bias. Statistical analyses were done using Comprehensive Meta Analysis software version 3 (Biostat).

## Results

The 74 studies included in this review involved unique 10 444 adult participants (modal sample size: n = 120) receiving MOUD, with 60 studies eligible for meta-analyses (n = 7000; modal sample size: n = 40). Studies were published between 1984 and 2019.

### Psychomotor Stimulants

Twenty-two studies tested the efficacy of contingency management for increasing abstinence from psychomotor stimulant use, with 18 (82%) reporting significant increases in abstinence at end-of-treatment assessment (eTable 1 in the Supplement). Participants were treated with methadone as the MOUD in all but 1 study (21 of 22 [95%]). The mean (SD) contingency management duration was 17.2 (13.8) weeks, and the mean (SD) maximum daily earnings was $14.51 ($11.94).

**Figure 2.**
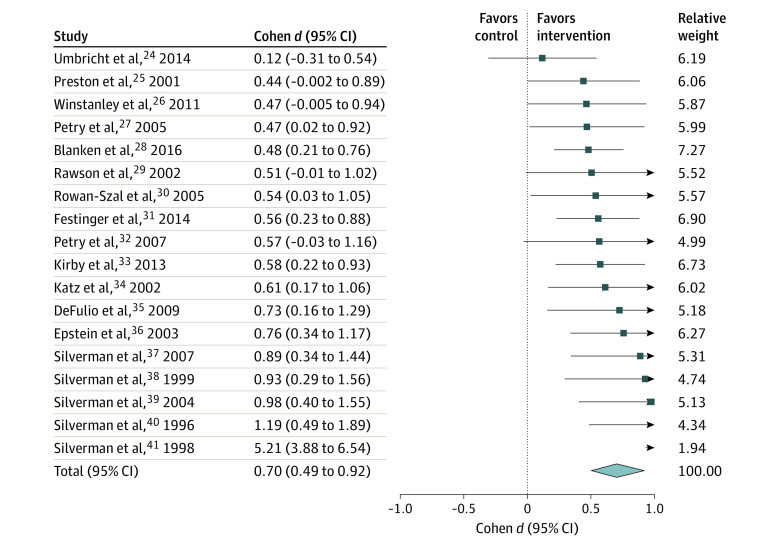
Forest Plot of Treatment Effect Sizes of Contingency Management vs Controls: Abstinence From Psychomotor Stimulant Use

There was sufficient information to calculate effect sizes for 18 of 22 studies (81.8%) (Figure 2).^24,25,26,27,28,29,30,31,32,33,34,35,36,37,38,39,40,41^ Contingency management was associated with an overall medium-large effect size on abstinence compared with controls at the end-of-treatment assessment (Cohen *d* = 0.70; 95% CI, 0.49-0.92; *I^2^* = 71.8%).

### Polysubstance Use

Twenty-three studies tested the efficacy of contingency management for increasing abstinence from 2 or more drugs. Contingency management was associated with increased abstinence at the end-of-treatment assessment in 16 studies (70%) (eTable 2 in the Supplement). Notably, psychomotor stimulants were among the drugs targeted in all of these studies.

MOUD type varied across studies; methadone was prescribed in 13 (57%), buprenorphine in 6 (26%), naltrexone in 2 (9%), levacetylmethadol in 1 (4%), and combined methadone and buprenorphine in 2 (9%). The mean (SD) contingency management duration was 14.3 (7.0) weeks, and the mean (SD) maximum daily earnings was $10.63 ($7.45).

**Figure 3.**
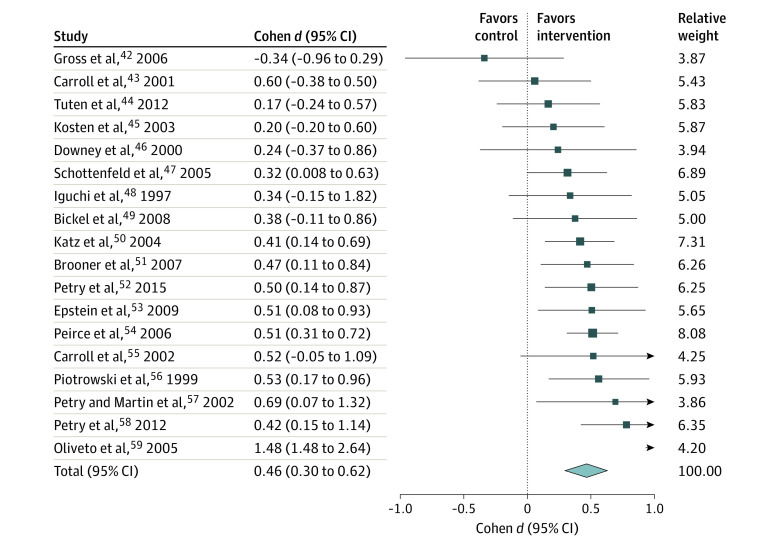
Forest Plot of Treatment Effect Sizes of Contingency Management vs Controls: Abstinence From Polysubstance Use

There was sufficient information to calculate effect sizes for 18 of 23 studies (78%) (Figure 3).^42,43,44,45,46,47,48,49,50,51,52,53,54,55,56,57,58,59^ Contingency management was associated with an overall small-medium effect size on abstinence compared with controls at the end-of-treatment assessment (Cohen *d =* 0.46; 95% CI, 0.30-0.62; *I^2^* = 64.8%).

### Illicit Opioids

Eleven studies tested the efficacy of contingency management for increasing abstinence from illicit opioid use. Contingency management was associated with increased abstinence at the end of treatment in 7 studies (64%) (eTable 3 in the Supplement).

Methadone was prescribed in 9 studies (82%), buprenorphine in 1 (9%), and naltrexone in 1 (9%). The mean (SD) contingency management duration was 13.9 (7.2) weeks, and the mean (SD) maximum daily earnings was $10.25 ($5.32).

**Figure 4.**
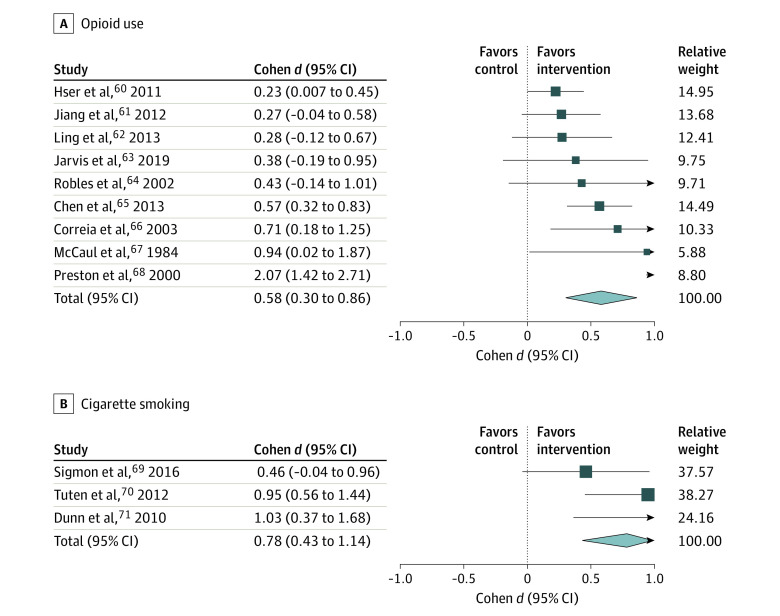
Forest Plots of Treatment Effect Sizes of Contingency Management vs Controls: Abstinence From Illicit Opioid Use and Cigarette Smoking

There was sufficient information to calculate effect sizes for 9 studies (82%) (Figure 4A).^60,61,62,63,64,65,66,67,68^ Contingency management again was associated with a medium-large effect size on abstinence compared with controls at the end-of-treatment assessment (Cohen *d* = 0.58; 95% CI, 0.30-0.86; *I^2^* = 75.9%).

### Cigarette Smoking

Five studies tested the efficacy of contingency management for increasing abstinence from cigarette smoking. Contingency management was associated with increased abstinence in 4 studies (eTable 4 in the Supplement).

Methadone was the MOUD prescribed in all studies, with 2 involving both methadone and buprenorphine. The mean (SD) contingency management duration was 7.6 (5.2) weeks. The mean (SD) maximum daily earnings was $15.09 ($10.01).

There was sufficient information to calculate effect sizes for 3 studies (Figure 4B).^69,70,71^ Contingency management was associated with an overall medium-large effect size with contingency management increasing abstinence compared with controls at the end-of-treatment assessment (Cohen *d* = 0.78; 95% CI, 0.43-1.14; *I^2^* = 21.4%).

### Therapy Attendance

Eleven studies tested the efficacy of contingency management for increasing therapy attendance (eTable 5A in the Supplement). Contingency management was associated with increased therapy attendance in 5 studies (45%).

Studies that targeted therapy attendance and drug abstinence are also included in eTables 1 to 3 in the Supplement. All studies examining therapy attendance prescribed methadone as the MOUD. The mean (SD) contingency management duration was 11.3 (6.0) weeks, and the mean (SD) maximum daily earnings was $11.18 ($12.60).

**Figure 5.**
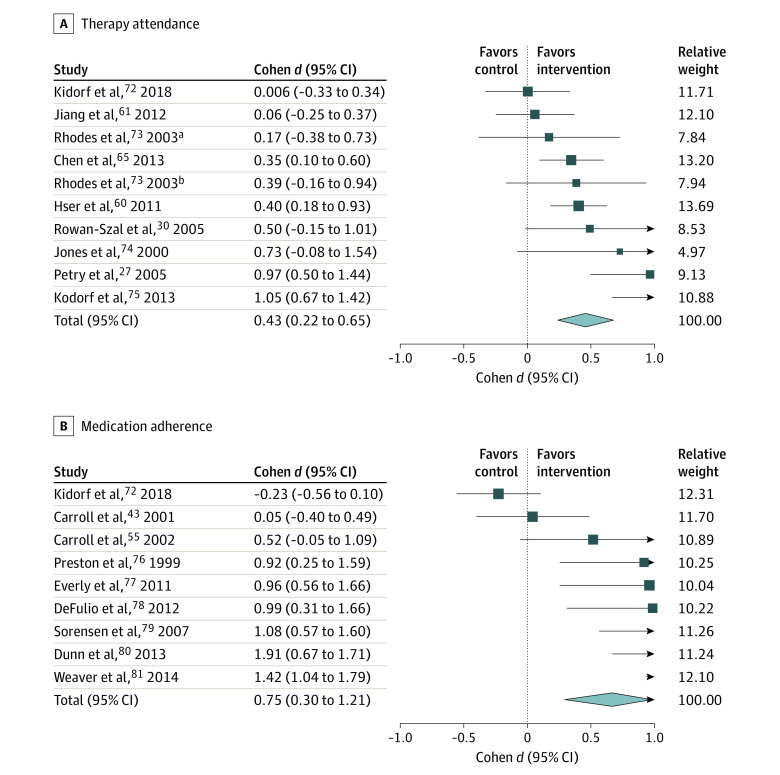
Forest Plots of Treatment Effect Sizes of Contingency Management vs Controls: Therapy Attendance and Medication Adherence ^a^Experiment 1. ^b^Experiment 2.

There was sufficient information to calculate effect sizes for 10 studies (91%) (Figure 5A).^27,30,60,61,65,72,73,74,75^ Contingency management was associated with an overall small-medium effect size on increasing therapy attendance compared with controls (Cohen *d* = 0.43; 95% CI, 0.22-0.65; *I^2^* = 68.6%).

### Medication Adherence

The 9 studies that tested contingency management for medication adherence are displayed in eTable 5B in the Supplement. Contingency management was associated with increased medication adherence in 6 studies.

Six of 9 targeted naltrexone adherence, 1 targeted methadone adherence, and 2 targeted adherence to other medications. Two studies that tested medication adherence also targeted polydrug abstinence, and 1 targeted counseling attendance. The mean (SD) contingency management duration was 17.4 (7.2) weeks, and the mean (SD) maximum daily earnings was $10.43 ($5.77).

There was sufficient information to calculate effect sizes for all studies in this category (Figure 5B).^43,55,72,76,77,78,79,80,81^ Contingency management was associated with an overall medium-large effect size compared with controls (Cohen *d* = 0.75; 95% CI, 0.30-1.21; *I^2^* = 69.2%).

### Pooled Abstinence and Treatment Adherence Effect Sizes

When combining across all trials examining abstinence as an outcome, contingency management was associated with significant increased abstinence compared with control (Cohen *d* = 0.58; 95% CI, 0.47-0.69; *I^2^* = 69.2%) (eFigure 1 in the Supplement). Similarly, contingency management was associated with increased treatment adherence when studies examining therapy attendance and medication adherence were combined (Cohen *d* = 0.62; 95% CI, 0.40-0.84; *I^2^* = 78.9%) (eFigure 2 in the Supplement).

### Moderator Analysis of Pooled Abstinence and Treatment Adherence Effect Sizes

Moderator analyses of maximum daily earnings, contingency management duration in weeks, and quality score (without blinding) (eTables 1-5 in the Supplement) were conducted for pooled abstinence effect sizes (eFigure 1 in the Supplement) and pooled treatment adherence effect sizes (eFigure 2 in the Supplement). In both analyses, the only significant moderator was maximum daily earnings (pooled abstinence effect sizes: *Q* = 5.67, *P* = .02; pooled treatment adherence effect sizes: *Q* = 4.82, *P* = .03), corresponding with a significant positive association between maximum daily earnings and effect size. Detailed information on this assessment can be found in eTable 8 in the Supplement.

### Pooled Follow-up Effect Sizes

Follow-up effect sizes were obtained for only 7 of 74 studies (9%), 6 of which examined abstinence from drug use and 1, naltrexone adherence. Given this relatively small number, we combined trials across outcomes (eFigure 3 in the Supplement). The pooled effect size was not significant (Cohen *d* = 0.02; 95% CI, –0.16 to 0.21; *I^2^* = 27.0%), indicating a lack of treatment effect after contingency management discontinuation.

### Quality Assessment and Publication Bias

In our quality assessment excluding the blinding component, 37 of 71 studies (52%) were rated strong, 26 (37%) were rated moderate, and 8 (11%) were rated weak. The modal score for each targeted outcome was strong with 2 exceptions: polysubstance use and medication adherence. For both study categories, the modal score was moderate. When the blinding component was included, the overall modal score was moderate (34 of 71 [48%]). Methodological weaknesses that led to study quality scores of moderate or weak often included insufficient or poor information on possible selection bias and/or withdrawal and dropout data. Individual scores for each component and global scores for each study are detailed in eTable 6 in the Supplement.

A funnel plot was created with all studies included in the meta-analyses to assess for publication bias. Funnel plots display individual study effect estimates against their precision, with a greater degree of asymmetry suggesting a greater possibility of publication bias. Examination of the funnel plot (eFigure 4 in the Supplement) showed no conclusive indication of publication bias. We tested study sample size as a moderator of effect size to further examine for publication bias and found no evidence that effect size was significantly associated with sample size (*Q* = 2.26, *P* = .13).

## Discussion

This systematic review and meta-analysis provides support for the efficacy of contingency management for addressing a wide range of substantive clinical problems common among people receiving MOUD, including the current crisis of comorbid stimulant use disorder. The meta-analysis demonstrated significant associations across all 6 clinical problems examined. Of interest, 18 of 22 studies (81.8%) reviewed reported statistically significant effects of contingency management on abstinence from psychomotor stimulant use, with a medium-large pooled effect size (Cohen *d* = 0.70; 95% CI, 0.49-0.92). That effect size translates to 75.8% of those treated with contingency management having an outcome superior to the mean outcome in the control condition.^82^ This is especially notable because contingency management is the only intervention that has reliably increased abstinence from psychomotor stimulants in randomized clinical trials across more than 30 years of research.^12^ Psychomotor stimulant use among those with MOUD has reached a crisis level that demands attention owing to its role in fatal overdose.^5,8,9^ That said, psychomotor stimulant use is by no means the only pressing problem nor only potentially fatal problem in the MOUD population. Our review supports the association of contingency management with an increase in abstinence from illicit opioid use at an effect size of 0.58, which translates to 71.9% of patients treated with contingency management having outcomes superior to the mean outcome of the control interventions. This observation stands in contrast to findings from a 2017 contingency management review^14^ reporting negative results for illicit opioids, but that review only included 2 studies examining opioids while the present review included 11, providing greater statistical power to discern an effect. A substantive threat to the efficacy of MOUD is poor adherence, and this review illustrates a medium-large effect size (Cohen *d* = 0.75) for medication adherence, with 7 of 9 studies examining MOUD adherence specifically. That effect size translates to 77.3% of patients treated with contingency management having outcomes superior to the mean outcomes of the control interventions. This meta-analysis supports findings that contingency management is also efficacious for various other clinical concerns common among those receiving MOUD with Cohen *d*’s ranging from 0.43 for therapy attendance to 0.78 for cigarette smoking underscoring the breadth of contingency management’s efficacy in this population, effect sizes that translate to 66.6% and 78.2% of patients treated with contingency management having outcomes superior to the mean outcomes of the control interventions. Overall, this evidence suggests that contingency management has potential to produce broad, substantive improvements in outcomes among patients receiving MOUD.

One observation that warrants underscoring is that among studies targeting abstinence from substance use, the smallest overall effect size was observed with polysubstance abstinence (Cohen *d* = 0.46). It appears that when the number of drugs simultaneously targeted by contingency management increased, the overall effect size decreased, although remaining statistically significant. This pattern is consistent with results from prior reviews^14^ and cautions against including too many targets simultaneously without making adjustments to the intervention (eg, increasing the monetary value of the incentive accordingly). Similarly, our finding that contingency management was most effective when greater maximum daily earnings were offered is consistent with prior research^22^ and demonstrates the importance of adequate financial incentives in contingency management interventions.

The follow-up results in the present meta-analysis are also consistent with those from a prior meta-analysis,^14^ showing that treatment effects often dissipate after contingency management is discontinued. This is not surprising and is consistent with other maintenance therapies for other chronic medical conditions.^83^ Nevertheless, relapse prevention after contingency management discontinuation needs to be considered in the treatment planning process. Two evidence-based options are to combine contingency management with other psychosocial interventions, such as Community Reinforcement Approach therapy, that help to assure that naturalistic sources of reinforcement for sober living are in place prior to discontinuing contingency management^84^ or enroll patients in longer-term (ie, what might be deemed maintenance) contingency management interventions like the Therapeutic Workplace.^85^ Our findings demonstrate that additional research on effective strategies for sustaining longer-term abstinence from drug use with contingency management interventions are sorely needed. Importantly, this situation is more a failure to explicitly examine efficacious strategies for sustaining behavior change rather than one where many strategies have been examined and failed.

The results of this meta-analysis have important implications for public health officials and clinicians. Primarily, they demonstrate that contingency management may be efficacious in treating a wide range of substantive clinical problems common among patients receiving MOUD. Nevertheless, there remains a long-standing challenge of increasing use of contingency management in community treatment clinics. The most substantial obstacle in the US is the reluctance of the Centers for Medicare & Medicaid Services (CMS) to allow Medicaid funds to be used for contingency management out of concern for potential fraud.^86^ However, it is important to underscore that, to our knowledge, there are no federal legal constraints against using Medicaid funds for contingency management nor any cases in which contingency management was associated with Medicaid fraud. Indeed, the Affordable Care Act mandated that CMS investigate the use of contingency management for treating a wide range of behavioral health conditions.^87^ Perhaps not surprisingly, the strongest evidence from the Affordable Care Act investigation was on contingency management’s effectiveness in treating substance use disorders, including smoking cessation among pregnant and newly postpartum individuals.^88^ We do not debate that safeguards are needed to protect against fraud, but we know of no evidence linking contingency management to fraud nor suggesting that contingency management is any more likely to result in fraud than other CMS-supported medical services. Given the overwhelming evidence supporting contingency management’s efficacy, we believe a more prudent approach by the CMS Office of Inspector General would be to support efforts to develop best practices for incorporating contingency management into MOUD therapeutic protocols and actively monitor against fraud using existing or new monitoring systems (eg, the Healthcare Fraud Prevention Partnership^89^). This is especially important for patients receiving MOUD who have potentially fatal conditions, such as stimulant use disorder, for which contingency management is the only efficacious intervention.

Beyond obtaining CMS support, there is a related practical need for development of materials and venues for training in contingency management clinical best practices.^90^ That will likely require federal support and thoughtful review of existing resources and implementation efforts, such as published therapist manuals and books on contingency management for treating substance use disorders and the World Bank’s Conditional Cash Transfer program that leveraged the contingency management scientific foundation in developing their global antipoverty programs.^91,92,93^

### Limitations

Limitations of this meta-analysis include the limited number of studies that completed posttreatment follow-up assessments after contingency management discontinuation. Again, we deem longer-term maintenance of treatment effects a critical gap for future contingency management research. Additionally, the included studies measured outcomes using a number of different definitions of abstinence (eg, longest duration, proportion of negative urine test results), which may have increased heterogeneity. Similarly, studies in the area of therapeutic attendance used varied outcomes (eg, percent of patients retained, number of sessions attended), as did those assessing medication adherence (eg, number of naltrexone doses accepted, completing hepatitis B vaccination). Of note, the results from the meta-analysis remained significant despite such variability, which suggests convergent validity across the various outcome measures. Additionally, the MOUD dose received by patients often varied across studies. Adequate dose is a critical concern in MOUD because an insufficient dose may increase the odds of relapse^94^; this is of particular relevance to those studies testing contingency management for increasing abstinence from illicit opioids (eTable 3 in the Supplement), but treatment in other areas may be undermined by ongoing illicit opioid use as well (eg, treatment retention, eTable 5A in the Supplement). Further, most studies in this review involved patients receiving methadone. Future research is needed with more widely available MOUD, namely buprenorphine but also naltrexone.^95^

## Conclusions

This systematic review and meta-analysis underscores the association of contingency management with treatment of a wide range of clinical problems common among patients receiving MOUD. The results support a position that policy makers including CMS should make concerted efforts to support broad dissemination of contingency management to the many community clinics throughout the US currently struggling with the challenges of the opioid crisis, especially concomitant psychomotor stimulant use among patients taking MOUD.

## Notes

Supplement. eTable 1. Studies examining abstinence from stimulants (n=22) eTable 2. Studies examining abstinence from multiple substances (n=23) eTable 3. Studies examining abstinence from illicit opioids (n=11) eTable 4. Studies targeting abstinence from cigarette smoking (n=5) eTable 5. Studies targeting therapy attendance or medication adherence outcomes (n=18) eTable 6. Detailed quality assessment data that were gathered using the Effective Public Health Practice Project tool (Thomas et al., 2004) eTable 7. Studies excluded at full-text review with reasons for exclusion eTable 8. Moderator analysis results eFigure 1. Forest plot demonstrating individual and overall Cohen’s d for all studies that targeted abstinence from substances as outcomes eFigure 2. Forest plot demonstrating individual and overall Cohen’s d for all studies that targeted treatment adherence (i.e., therapy attendance or medication adherence) as outcomes eFigure 3. Forest plot demonstrating the effect size for the seven studies for which we could calculate follow-up effect sizes eFigure 4. Funnel plot assessing for publication bias among studies included in the meta-analysis eReferences.

## References

[1] Substance Abuse and Mental Health Services Administration. 2019 NSDUH Annual National Report. Published September 11, 2020. Accessed April 6, 2021. https://www.samhsa.gov/data/report/2019-nsduh-annual-national-report

[2] Scholl L, Seth P, Kariisa M, Wilson N, Baldwin G. Drug and opioid-involved overdose deaths: United States, 2013-2017. MMWR Morb Mortal Wkly Rep. 2018;67(5152):1419-1427. doi:10.15585/mmwr.mm675152e1 30605448 PMC6334822

[3] Woolf SH, Schoomaker H. Life expectancy and mortality rates in the United States, 1959-2017. JAMA. 2019;322(20):1996-2016. doi:10.1001/jama.2019.16932 31769830 PMC7146991

[4] Murphy SM. The cost of opioid use disorder and the value of aversion. Drug Alcohol Depend. 2020;217:108382. doi:10.1016/j.drugalcdep.2020.108382 33183909 PMC7737485

[5] Cicero TJ, Ellis MS, Kasper ZA. Polysubstance use: a broader understanding of substance use during the opioid crisis. Am J Public Health. 2020;110(2):244-250. doi:10.2105/AJPH.2019.305412 31855487 PMC6951387

[6] Mattick RP, Breen C, Kimber J, Davoli M. Buprenorphine maintenance versus placebo or methadone maintenance for opioid dependence. Cochrane Database Syst Rev. 2014;(2):CD002207. doi:10.1002/14651858.CD002207.pub4 24500948 PMC10617756

[7] Paulus MP, Stewart JL. Neurobiology, clinical presentation, and treatment of methamphetamine use disorder: a review. JAMA Psychiatry. 2020;77(9):959-966. doi:10.1001/jamapsychiatry.2020.0246 32267484 PMC8098650

[8] Tsui JI, Mayfield J, Speaker EC, . Association between methamphetamine use and retention among patients with opioid use disorders treated with buprenorphine. J Subst Abuse Treat. 2020;109:80-85. doi:10.1016/j.jsat.2019.10.005 31810594

[9] National Institute on Drug Abuse. Overdose death rates. Published March 20, 2020. Accessed April 22, 2020. https://www.drugabuse.gov/related-topics/trends-statistics/overdose-death-rates

[10] Trivedi MH, Walker R, Ling W, . Bupropion and naltrexone in methamphetamine use disorder. N Engl J Med. 2021;384(2):140-153. doi:10.1056/NEJMoa2020214 33497547 PMC8111570

[11] Brandt L, Chao T, Comer SD, Levin FR. Pharmacotherapeutic strategies for treating cocaine use disorder: what do we have to offer? Addiction. 2021;116(4):694-710. doi:10.1111/add.15242 32888245 PMC7930140

[12] De Crescenzo F, Ciabattini M, D’Alò GL, . Comparative efficacy and acceptability of psychosocial interventions for individuals with cocaine and amphetamine addiction: a systematic review and network meta-analysis. PLoS Med. 2018;15(12):e1002715. doi:10.1371/journal.pmed.1002715 30586362 PMC6306153

[13] Griffith JD, Rowan-Szal GA, Roark RR, Simpson DD. Contingency management in outpatient methadone treatment: a meta-analysis. Drug Alcohol Depend. 2000;58(1-2):55-66. doi:10.1016/S0376-8716(99)00068-X 10669055

[14] Ainscough TS, McNeill A, Strang J, Calder R, Brose LS. Contingency Management interventions for non-prescribed drug use during treatment for opiate addiction: a systematic review and meta-analysis. Drug Alcohol Depend. 2017;178:318-339. doi:10.1016/j.drugalcdep.2017.05.028 28688295 PMC5558146

[15] The Biden-Harris Administration’s Statement of Drug Policy Priorities for Year One. Released April 1, 2021. Accessed April 6, 2021. https://www.whitehouse.gov/wp-content/uploads/2021/03/BidenHarris-Statement-of-Drug-Policy-Priorities-April-1.pdf?fbclid=IwAR2TBk34U_XRqlqK_pAYnUd_9f7zY3IbCQI9KxI6S5eYeRJdFzl9B09hZ84Published

[16] WebPlotDigitizer. Accessed June 29, 2021. https://automeris.io/WebPlotDigitizer/

[17] Dunn K, DeFulio A, Everly JJ, . Employment-based reinforcement of adherence to oral naltrexone in unemployed injection drug users: 12-month outcomes. Psychol Addict Behav. 2015;29(2):270-276. doi:10.1037/adb0000010 25134047 PMC4339630

[18] DeFulio A, Silverman K. Employment-based abstinence reinforcement as a maintenance intervention for the treatment of cocaine dependence: post-intervention outcomes. Addiction. 2011;106(5):960-967. doi:10.1111/j.1360-0443.2011.03364.x 21226886 PMC3074032

[19] Kosten T, Poling J, Oliveto A. Effects of reducing contingency management values on heroin and cocaine use for buprenorphine- and desipramine-treated patients. Addiction. 2003;98(5):665-671. doi:10.1046/j.1360-0443.2003.00380.x 12751984

[20] Higgins ST, Heil SH, Dantona R, Donham R, Matthews M, Badger GJ. Effects of varying the monetary value of voucher-based incentives on abstinence achieved during and following treatment among cocaine-dependent outpatients. Addiction. 2007;102(2):271-281. doi:10.1111/j.1360-0443.2006.01664.x 17222282

[21] Thomas BH, Ciliska D, Dobbins M, Micucci S. A process for systematically reviewing the literature: providing the research evidence for public health nursing interventions. Worldviews Evid Based Nurs. 2004;1(3):176-184. doi:10.1111/j.1524-475X.2004.04006.x 17163895

[22] Cohen, J. Statistical Power Analysis for the Behavioral Sciences. 2nd ed. Academic Press; 1988.

[23] Lussier JP, Heil SH, Mongeon JA, Badger GJ, Higgins ST. A meta-analysis of voucher-based reinforcement therapy for substance use disorders. Addiction. 2006;101(2):192-203. doi:10.1111/j.1360-0443.2006.01311.x 16445548

[24] Umbricht A, DeFulio A, Winstanley EL, . Topiramate for cocaine dependence during methadone maintenance treatment: a randomized controlled trial. Drug Alcohol Depend. 2014;140:92-100. doi:10.1016/j.drugalcdep.2014.03.033 24814607 PMC4431633

[25] Preston KL, Umbricht A, Wong CJ, Epstein DH. Shaping cocaine abstinence by successive approximation. J Consult Clin Psychol. 2001;69(4):643-654. doi:10.1037/0022-006X.69.4.643 11550730

[26] Winstanley EL, Bigelow GE, Silverman K, Johnson RE, Strain EC. A randomized controlled trial of fluoxetine in the treatment of cocaine dependence among methadone-maintained patients. J Subst Abuse Treat. 2011;40(3):255-264. doi:10.1016/j.jsat.2010.11.010 21266301 PMC3078567

[27] Petry NM, Peirce JM, Stitzer ML, . Effect of prize-based incentives on outcomes in stimulant abusers in outpatient psychosocial treatment programs: a national drug abuse treatment clinical trials network study. Arch Gen Psychiatry. 2005;62(10):1148-1156. doi:10.1001/archpsyc.62.10.1148 16203960

[28] Blanken P, Hendriks VM, Huijsman IA, van Ree JM, van den Brink W. Efficacy of cocaine contingency management in heroin-assisted treatment: results of a randomized controlled trial. Drug Alcohol Depend. 2016;164:55-63. doi:10.1016/j.drugalcdep.2016.04.018 27177805

[29] Rawson RA, Huber A, McCann M, . A comparison of contingency management and cognitive-behavioral approaches during methadone maintenance treatment for cocaine dependence. Arch Gen Psychiatry. 2002;59(9):817-824. doi:10.1001/archpsyc.59.9.817 12215081

[30] Rowan-Szal GA, Bartholomew NG, Chatham LR, Simpson DD. A combined cognitive and behavioral intervention for cocaine-using methadone clients. J Psychoactive Drugs. 2005;37(1):75-84. doi:10.1080/02791072.2005.10399750 15916253

[31] Festinger DS, Dugosh KL, Kirby KC, Seymour BL. Contingency management for cocaine treatment: cash vs. vouchers. J Subst Abuse Treat. 2014;47(2):168-174. doi:10.1016/j.jsat.2014.03.001 24746956 PMC4504189

[32] Petry NM, Alessi SM, Hanson T, Sierra S. Randomized trial of contingent prizes versus vouchers in cocaine-using methadone patients. J Consult Clin Psychol. 2007;75(6):983-991. doi:10.1037/0022-006X.75.6.983 18085914

[33] Kirby KC, Carpenedo CM, Dugosh KL, . Randomized clinical trial examining duration of voucher-based reinforcement therapy for cocaine abstinence. Drug Alcohol Depend. 2013;132(3):639-645. doi:10.1016/j.drugalcdep.2013.04.015 23680075 PMC3770760

[34] Katz EC, Chutuape MA, Jones HE, Stitzer ML. Voucher reinforcement for heroin and cocaine abstinence in an outpatient drug-free program. Exp Clin Psychopharmacol. 2002;10(2):136-143. doi:10.1037/1064-1297.10.2.136 12022799

[35] DeFulio A, Donlin WD, Wong CJ, Silverman K. Employment-based abstinence reinforcement as a maintenance intervention for the treatment of cocaine dependence: a randomized controlled trial. Addiction. 2009;104(9):1530-1538. doi:10.1111/j.1360-0443.2009.02657.x 19686522 PMC2729763

[36] Epstein DH, Hawkins WE, Covi L, Umbricht A, Preston KL. Cognitive-behavioral therapy plus contingency management for cocaine use: findings during treatment and across 12-month follow-up. Psychol Addict Behav. 2003;17(1):73-82. doi:10.1037/0893-164X.17.1.73 12665084 PMC1224747

[37] Silverman K, Wong CJ, Needham M, . A randomized trial of employment-based reinforcement of cocaine abstinence in injection drug users. J Appl Behav Anal. 2007;40(3):387-410. doi:10.1901/jaba.2007.40-387 17970256 PMC1986688

[38] Silverman K, Chutuape MA, Bigelow GE, Stitzer ML. Voucher-based reinforcement of cocaine abstinence in treatment-resistant methadone patients: effects of reinforcement magnitude. Psychopharmacology (Berl). 1999;146(2):128-138. doi:10.1007/s002130051098 10525747

[39] Silverman K, Robles E, Mudric T, Bigelow GE, Stitzer ML. A randomized trial of long-term reinforcement of cocaine abstinence in methadone-maintained patients who inject drugs. J Consult Clin Psychol. 2004;72(5):839-854. doi:10.1037/0022-006X.72.5.839 15482042

[40] Silverman K, Higgins ST, Brooner RK, . Sustained cocaine abstinence in methadone maintenance patients through voucher-based reinforcement therapy. Arch Gen Psychiatry. 1996;53(5):409-415. doi:10.1001/archpsyc.1996.01830050045007 8624184

[41] Silverman K, Wong CJ, Umbricht-Schneiter A, Montoya ID, Schuster CR, Preston KL. Broad beneficial effects of cocaine abstinence reinforcement among methadone patients. J Consult Clin Psychol. 1998;66(5):811-824. doi:10.1037/0022-006X.66.5.811 9803700

[42] Gross A, Marsch LA, Badger GJ, Bickel WK. A comparison between low-magnitude voucher and buprenorphine medication contingencies in promoting abstinence from opioids and cocaine. Exp Clin Psychopharmacol. 2006;14(2):148-156. doi:10.1037/1064-1297.14.2.148 16756418

[43] Carroll KM, Ball SA, Nich C, . Targeting behavioral therapies to enhance naltrexone treatment of opioid dependence: efficacy of contingency management and significant other involvement. Arch Gen Psychiatry. 2001;58(8):755-761. doi:10.1001/archpsyc.58.8.755 11483141 PMC3651594

[44] Tuten M, Svikis DS, Keyser-Marcus L, O’Grady KE, Jones HE. Lessons learned from a randomized trial of fixed and escalating contingency management schedules in opioid-dependent pregnant women. Am J Drug Alcohol Abuse. 2012;38(4):286-292. doi:10.3109/00952990.2011.643977 22352784

[45] Kosten T, Oliveto A, Feingold A, . Desipramine and contingency management for cocaine and opiate dependence in buprenorphine maintained patients. Drug Alcohol Depend. 2003;70(3):315-325. doi:10.1016/S0376-8716(03)00032-2 12757969

[46] Downey KK, Helmus TC, Schuster CR. Treatment of heroin-dependent poly-drug abusers with contingency management and buprenorphine maintenance. Exp Clin Psychopharmacol. 2000;8(2):176-184. doi:10.1037/1064-1297.8.2.176 10843300

[47] Schottenfeld RS, Chawarski MC, Pakes JR, Pantalon MV, Carroll KM, Kosten TR. Methadone versus buprenorphine with contingency management or performance feedback for cocaine and opioid dependence. Am J Psychiatry. 2005;162(2):340-349. doi:10.1176/appi.ajp.162.2.340 15677600

[48] Iguchi MY, Belding MA, Morral AR, Lamb RJ, Husband SD. Reinforcing operants other than abstinence in drug abuse treatment: an effective alternative for reducing drug use. J Consult Clin Psychol. 1997;65(3):421-428. doi:10.1037/0022-006X.65.3.421 9170765

[49] Bickel WK, Marsch LA, Buchhalter AR, Badger GJ. Computerized behavior therapy for opioid-dependent outpatients: a randomized controlled trial. Exp Clin Psychopharmacol. 2008;16(2):132-143. doi:10.1037/1064-1297.16.2.132 18489017 PMC2746734

[50] Katz EC, Chutuape MA, Jones H, Jasinski D, Fingerhood M, Stitzer M. Abstinence incentive effects in a short-term outpatient detoxification program. Exp Clin Psychopharmacol. 2004;12(4):262-268. doi:10.1037/1064-1297.12.4.262 15571443

[51] Brooner RK, Kidorf MS, King VL, Stoller KB, Neufeld KJ, Kolodner K. Comparing adaptive stepped care and monetary-based voucher interventions for opioid dependence. Drug Alcohol Depend. 2007;88(suppl 2):S14-S23. doi:10.1016/j.drugalcdep.2006.12.006 PMC1948819 17257782

[52] Petry NM, Alessi SM, Barry D, Carroll KM. Standard magnitude prize reinforcers can be as efficacious as larger magnitude reinforcers in cocaine-dependent methadone patients. J Consult Clin Psychol. 2015;83(3):464-472. doi:10.1037/a0037888 25198284 PMC4362849

[53] Epstein DH, Schmittner J, Umbricht A, Schroeder JR, Moolchan ET, Preston KL. Promoting abstinence from cocaine and heroin with a methadone dose increase and a novel contingency. Drug Alcohol Depend. 2009;101(1-2):92-100. doi:10.1016/j.drugalcdep.2008.11.006 19101098 PMC2943844

[54] Peirce JM, Petry NM, Stitzer ML, . Effects of lower-cost incentives on stimulant abstinence in methadone maintenance treatment: a National Drug Abuse Treatment Clinical Trials Network study. Arch Gen Psychiatry. 2006;63(2):201-208. doi:10.1001/archpsyc.63.2.201 16461864

[55] Carroll KM, Sinha R, Nich C, Babuscio T, Rounsaville BJ. Contingency management to enhance naltrexone treatment of opioid dependence: a randomized clinical trial of reinforcement magnitude. Exp Clin Psychopharmacol. 2002;10(1):54-63. doi:10.1037/1064-1297.10.1.54 11866252

[56] Piotrowski NA, Tusel DJ, Sees KL, . Contingency contracting with monetary reinforcers for abstinence from multiple drugs in a methadone program. Exp Clin Psychopharmacol. 1999;7(4):399-411. doi:10.1037/1064-1297.7.4.399 10609975

[57] Petry NM, Martin B. Low-cost contingency management for treating cocaine- and opioid-abusing methadone patients. J Consult Clin Psychol. 2002;70(2):398-405. doi:10.1037/0022-006X.70.2.398 11952198

[58] Petry NM, Alessi SM, Ledgerwood DM. A randomized trial of contingency management delivered by community therapists. J Consult Clin Psychol. 2012;80(2):286-298. doi:10.1037/a0026826 22250852 PMC3725552

[59] Oliveto A, Poling J, Sevarino KA, . Efficacy of dose and contingency management procedures in LAAM-maintained cocaine-dependent patients. Drug Alcohol Depend. 2005;79(2):157-165. doi:10.1016/j.drugalcdep.2005.01.007 16002025

[60] Hser YI, Li J, Jiang H, . Effects of a randomized contingency management intervention on opiate abstinence and retention in methadone maintenance treatment in China. Addiction. 2011;106(10):1801-1809. doi:10.1111/j.1360-0443.2011.03490.x 21793958 PMC3174353

[61] Jiang H, Du J, Wu F, . Efficacy of contingency management in improving retention and compliance to methadone maintenance treatment: a random controlled study. Shanghai Arch Psychiatry. 2012;24(1):11-19. doi:10.3969/j.issn.1002-0829.2012.01.002 25324596 PMC4198887

[62] Ling W, Hillhouse M, Ang A, Jenkins J, Fahey J. Comparison of behavioral treatment conditions in buprenorphine maintenance. Addiction. 2013;108(10):1788-1798. doi:10.1111/add.12266 23734858 PMC3866908

[63] Jarvis BP, Holtyn AF, DeFulio A, . The effects of extended-release injectable naltrexone and incentives for opiate abstinence in heroin-dependent adults in a model therapeutic workplace: a randomized trial. Drug Alcohol Depend. 2019;197:220-227. doi:10.1016/j.drugalcdep.2018.12.026 30852374 PMC6440824

[64] Robles E, Stitzer ML, Strain EC, Bigelow GE, Silverman K. Voucher-based reinforcement of opiate abstinence during methadone detoxification. Drug Alcohol Depend. 2002;65(2):179-189. doi:10.1016/S0376-8716(01)00160-0 11772479

[65] Chen W, Hong Y, Zou X, McLaughlin MM, Xia Y, Ling L. Effectiveness of prize-based contingency management in a methadone maintenance program in China. Drug Alcohol Depend. 2013;133(1):270-274. doi:10.1016/j.drugalcdep.2013.05.028 23831409

[66] Correia CJ, Dallery J, Katz EC, Silverman K, Bigelow G, Stitzer ML. Single- versus dual-drug target: effects in a brief abstinence incentive procedure. Exp Clin Psychopharmacol. 2003;11(4):302-308. doi:10.1037/1064-1297.11.4.302 14599264

[67] McCaul ME, Stitzer ML, Bigelow GE, Liebson IA. Contingency management interventions: effects on treatment outcome during methadone detoxification. J Appl Behav Anal. 1984;17(1):35-43. doi:10.1901/jaba.1984.17-35 6725168 PMC1307916

[68] Preston KL, Umbricht A, Epstein DH. Methadone dose increase and abstinence reinforcement for treatment of continued heroin use during methadone maintenance. Arch Gen Psychiatry. 2000;57(4):395-404. doi:10.1001/archpsyc.57.4.395 10768702

[69] Sigmon SC, Miller ME, Meyer AC, . Financial incentives to promote extended smoking abstinence in opioid-maintained patients: a randomized trial. Addiction. 2016;111(5):903-912. doi:10.1111/add.13264 26638126 PMC4826799

[70] Tuten M, Fitzsimons H, Chisolm MS, Nuzzo PA, Jones HE. Contingent incentives reduce cigarette smoking among pregnant, methadone-maintained women: results of an initial feasibility and efficacy randomized clinical trial. Addiction. 2012;107(10):1868-1877. doi:10.1111/j.1360-0443.2012.03923.x 22716774 PMC3439534

[71] Dunn KE, Sigmon SC, Reimann EF, Badger GJ, Heil SH, Higgins ST. A contingency-management intervention to promote initial smoking cessation among opioid-maintained patients. Exp Clin Psychopharmacol. 2010;18(1):37-50. doi:10.1037/a0018649 20158293 PMC3605744

[72] Kidorf M, Brooner RK, Leoutsakos JM, Peirce J. Treatment initiation strategies for syringe exchange referrals to methadone maintenance: a randomized clinical trial. Drug Alcohol Depend. 2018;187:343-350. doi:10.1016/j.drugalcdep.2018.03.009 29709732

[73] Rhodes GL, Saules KK, Helmus TC, . Improving on-time counseling attendance in a methadone treatment program: a contingency management approach. Am J Drug Alcohol Abuse. 2003;29(4):759-773. doi:10.1081/ADA-120026259 14713138

[74] Jones HE, Haug NA, Stitzer ML, Svikis DS. Improving treatment outcomes for pregnant drug-dependent women using low-magnitude voucher incentives. Addict Behav. 2000;25(2):263-267. doi:10.1016/S0306-4603(98)00119-1 10795950

[75] Kidorf M, Brooner RK, Gandotra N, . Reinforcing integrated psychiatric service attendance in an opioid-agonist program: a randomized and controlled trial. Drug Alcohol Depend. 2013;133(1):30-36. doi:10.1016/j.drugalcdep.2013.06.005 23866988 PMC3786041

[76] Preston KL, Silverman K, Umbricht A, DeJesus A, Montoya ID, Schuster CR. Improvement in naltrexone treatment compliance with contingency management. Drug Alcohol Depend. 1999;54(2):127-135. doi:10.1016/S0376-8716(98)00152-5 10217552

[77] Everly JJ, DeFulio A, Koffarnus MN, . Employment-based reinforcement of adherence to depot naltrexone in unemployed opioid-dependent adults: a randomized controlled trial. Addiction. 2011;106(7):1309-1318. doi:10.1111/j.1360-0443.2011.03400.x 21320227 PMC3107896

[78] DeFulio A, Everly JJ, Leoutsakos JM, . Employment-based reinforcement of adherence to an FDA approved extended release formulation of naltrexone in opioid-dependent adults: a randomized controlled trial. Drug Alcohol Depend. 2012;120(1-3):48-54. doi:10.1016/j.drugalcdep.2011.06.023 21782353 PMC3245785

[79] Sorensen JL, Haug NA, Delucchi KL, . Voucher reinforcement improves medication adherence in HIV-positive methadone patients: a randomized trial. Drug Alcohol Depend. 2007;88(1):54-63. doi:10.1016/j.drugalcdep.2006.09.019 17056206 PMC1976289

[80] Dunn KE, Defulio A, Everly JJ, . Employment-based reinforcement of adherence to oral naltrexone treatment in unemployed injection drug users. Exp Clin Psychopharmacol. 2013;21(1):74-83. doi:10.1037/a0030743 23205722 PMC3641088

[81] Weaver T, Metrebian N, Hellier J, . Use of contingency management incentives to improve completion of hepatitis B vaccination in people undergoing treatment for heroin dependence: a cluster randomised trial. Lancet. 2014;384(9938):153-163. doi:10.1016/S0140-6736(14)60196-3 24725468

[82] Magnusson K. Interpreting Cohen’s d effect size: an interactive visualization. R Psychologist. Accessed May 21, 2021. https://rpsychologist.com/cohend/

[83] McLellan AT, Lewis DC, O’Brien CP, Kleber HD. Drug dependence, a chronic medical illness: implications for treatment, insurance, and outcomes evaluation. JAMA. 2000;284(13):1689-1695. doi:10.1001/jama.284.13.1689 11015800

[84] Higgins ST, Wong CJ, Badger GJ, Ogden DE, Dantona RL. Contingent reinforcement increases cocaine abstinence during outpatient treatment and 1 year of follow-up. J Consult Clin Psychol. 2000;68(1):64-72. doi:10.1037/0022-006X.68.1.64 10710841

[85] Silverman K, DeFulio A, Sigurdsson SO. Maintenance of reinforcement to address the chronic nature of drug addiction. Prev Med. 2012;55(suppl):S46-S53. doi:10.1016/j.ypmed.2012.03.013 22668883 PMC3437006

[86] Office of Inspector General (OIG), HHS. Medicare and state health care programs: fraud and abuse; revisions to the safe harbors under the anti-kickback statute and civil monetary penalty rules regarding beneficiary inducements: final rule. Fed Regist. 2016;81(235):88368-88409. 27992158

[87] US Department of Health & Human Services. About the Affordable Care Act. Last updated March 23, 2021. Accessed January 15, 2021. https://www.hhs.gov/healthcare/about-the-aca/index.html

[88] Baker TB, Fraser DL, Kobinsky K, . A randomized controlled trial of financial incentives to low income pregnant women to engage in smoking cessation treatment: effects on post-birth abstinence. J Consult Clin Psychol. 2018;86(5):464-473. doi:10.1037/ccp0000278 29389142

[89] Centers for Medicare & Medicaid Services. About the partnership. Updated April 5, 2021. Accessed April 1, 2021. https://www.cms.gov/hfpp/about

[90] Oluwoye O, Kriegel L, Alcover KC, McPherson S, McDonell MG, Roll JM. The dissemination and implementation of contingency management for substance use disorders: a systematic review. Psychol Addict Behav. 2020;34(1):99-110. doi:10.1037/adb0000487 31259569 PMC6938576

[91] Budney AJ, Higgins, ST. A Community Reinforcement Plus Vouchers Approach: Treating Cocaine Addiction. National Institute on Drug Abuse; 1998.

[92] Petry NM. Contingency Management for Substance Abuse Treatment: a Guide to Implementing This Evidence-Based Practice. Routledge; 2011.

[93] Ranganathan M, Lagarde M. Promoting healthy behaviours and improving health outcomes in low and middle income countries: a review of the impact of conditional cash transfer programmes. Prev Med. 2012;55(suppl):S95-S105. doi:10.1016/j.ypmed.2011.11.015 22178043

[94] National Academies of Sciences, Engineering, and Medicine; Health and Medicine Division; Board on Health Sciences Policy; Committee on Medication-Assisted Treatment for Opioid Use Disorder. Leshner AI, Mancher M, Eds. Medications for Opioid Use Disorder Save Lives. National Academies Press; 2019. doi:10.17226/25310 30896911

[95] Key substance use and mental health indicators in the United States: results from the 2019 National Survey on Drug Use and Health. Accessed June 28, 2021. https://www.samhsa.gov/data/sites/default/files/reports/rpt29393/2019NSDUHFFRPDFWHTML/2019NSDUHFFR090120.htm

