# Supplementary material for: Contingency Management for Patients Receiving Medication for Opioid Use Disorder: A Systematic Review and Meta-analysis
Source: JAMA Psychiatry. 2021 Aug 4;78(10):1–11. doi: 10.1001/jamapsychiatry.2021.1969 (PMC8340014; doi:10.1001/jamapsychiatry.2021.1969)
Supplement: Supplement. — eTable 1. Studies examining abstinence from stimulants (n=22) eTable 2. Studies examining abstinence from multiple substances (n=23) eTable 3. Studies examining abstinence from illicit opioids (n=11) eTable 4. Studies targeting abstinence from cigarette smoking (n=5) eTable 5. Studies targeting therapy attendance or medication adherence outcomes (n=18) eTable 6. Detailed quality assessment data that were gathered using the Effective Public Health Practice Project tool (Thomas et al., 2004) eTable 7. Studies excluded at full-text review with reasons for exclusion eTable 8. Moderator analysis results eFigure 1. Forest plot demonstrating individual and overall Cohen’s d for all studies that targeted abstinence from substances as outcomes eFigure 2. Forest plot demonstrating individual and overall Cohen’s d for all studies that targeted treatment adherence (i.e., therapy attendance or medication adherence) as outcomes eFigure 3. Forest plot demonstrating the effect size for the seven studies for which we could calculate follow-up effect sizes eFigure 4. Funnel plot assessing for publication bias among studies included in the meta-analysis eReferences. [file jamapsychiatry-e211969-s001.pdf]

Supplemental Online Content

Bolívar HA, Klemperer EM, Coleman SRM, DeSarno M, Skelly JM, Higgins ST. Contingency management for patients receiving medication for opioid use disorder: a systematic review and meta-analysis. *JAMA Psychiatry*. Published online August 4, 2021. doi:10.1001/jamapsychiatry.2021.1969

eTable 1. Studies examining abstinence from stimulants (n=22)
eTable 2. Studies examining abstinence from multiple substances (n=23)
eTable 3. Studies examining abstinence from illicit opioids (n=11)
eTable 4. Studies targeting abstinence from cigarette smoking (n=5)
eTable 5. Studies targeting therapy attendance or medication adherence outcomes (n=18)
eTable 6. Detailed quality assessment data that were gathered using the Effective Public Health Practice Project tool (Thomas et al., 2004)
eTable 7. Studies excluded at full-text review with reasons for exclusion
eTable 8. Moderator analysis results
eFigure 1. Forest plot demonstrating individual and overall Cohen's d for all studies that targeted abstinence from substances as outcomes
eFigure 2. Forest plot demonstrating individual and overall Cohen's d for all studies that targeted treatment adherence (i.e., therapy attendance or medication adherence) as outcomes
eFigure 3. Forest plot demonstrating the effect size for the seven studies for which we could calculate follow-up effect sizes
eFigure 4. Funnel plot assessing for publication bias among studies included in the meta-analysis
eReferences

This supplemental material has been provided by the authors to give readers additional information about their work.

© 2021 Bolívar HA et al. *JAMA Psychiatry.*

eTable 1. Studies examining abstinence from stimulants (n=22).

Author	Year	N	Study design	MOUD	CM duration (weeks)	Maximum earnings/day ($)	Conditions Compared	Outcomes	Statistically significant effect at end of Tx?	QA Rating[a]
Silverman et al.[1]	1996a	37	RCT	M	12	13.75	Contingent vouchers vs. Noncontingent vouchers[b] (yoked)	LDA	Yes	1
Silverman et al.[2]	1998	59	RCT	M	12	23.21	Escalating contingent vouchers with bonuses vs. Noncontingent vouchers with bonuses (yoked)	% cocaine abstinent	Yes	3
Silverman et al.[3]	1999	29	Within-subject	M	9	30.65[c]	Contingent vouchers vs. No voucher baseline	% negative samples	Yes	1
Robles et al.[4,d]	2000	72	Within-subject	M	0.3	50.00	Contingent vouchers vs. No voucher baseline	% negative samples	Yes	1
Preston et al.[5,e]	2001	80	RCT	M	12	4.29	Contingent vouchers vs. Noncontingent vouchers (yoked)	LDA	No	2
Katz et al.[6,d]	2002	40	Within-subject	M	1.6	36.36	Contingent vouchers vs. No vouchers	LDA	Yes	2
Rawson et al.[7]	2002	120	RCT	M	16	11.41	Contingent vouchers (with and without CBT) vs. No vouchers (or CBT alone)	% continuously cocaine abstinent > 3 weeks	Yes	1
Epstein et al.[8]	2003	193	RCT	M	12	13.75	Contingent vouchers vs. Noncontingent vouchers (yoked)	LDA	Yes	1

© 2021 Bolívar HA et al. *JAMA Psychiatry.*

Sigmon et al.[9]	2004	46	RCT	M	24	7.14	Contingent vouchers vs. No vouchers	% cocaine-negative samples	Yes	1
Silverman et al.[10]	2004	78	RCT	M	52	15.93	Contingent vouchers vs. No vouchers	% negative samples	Yes	1
Rowan-Szal et al.[11]	2005	61	RCT	M	8	0.45	Contingent vouchers vs. No vouchers	% continuously cocaine abstinent	Yes	2
Petry et al.[12]	2005	77	RCT	M	12	CNBD	Contingent vouchers vs. No vouchers	LDA	Yes	1
Silverman et al.[13]	2007	56	RCT	M	26	CNBD	Contingent access to work vs. Noncontingent access to work	% cocaine-negative samples	Yes	1
Petry et al.[14]	2007	76	RCT	M	12	5.27	Contingent vouchers or prizes vs. No vouchers	% of participants achieving continuous abstinence	Yes	2
Vandrey et al.[15]	2007	12	Within-subject	M	5.7	8.77	Contingent vouchers or cash vs. No vouchers or cash	% continuously cocaine abstinent	No	2
Defulio et al.[16,f]	2009	51	RCT	M	52	CNBD	Contingent access to work vs. Noncontingent access to work	% cocaine-negative samples	Yes	1
Winstanley et al.[17]	2011	145	RCT	M	12	13.75	Contingent vouchers + placebo or fluoxetine vs. Placebo or fluoxetine only	% cocaine-negative samples	No	2
Kirby et al.[18]	2013	130	RCT	M	36	CNBD	Contingent vouchers vs. Aftercare[g]	LDA	Yes	1

© 2021 Bolívar HA et al. *JAMA Psychiatry.*

Kennedy et al.[19]	2013	58	RCT	M	16	12.66	Contingent vouchers vs. Noncontingent vouchers	% samples negative for cocaine	Yes	1
Festinger et al.[20]	2014	222	RCT	M	12	CNBD	Contingent vouchers or cash vs. No vouchers or cash	LDA	Yes	2
Umbricht et al.[21]	2014	171	RCT	M	12	13.75	Contingent vouchers + TOP or placebo vs. Noncontingent vouchers (yoked) + TOP or placebo	LDA	No	1
Blanken et al.[22]	2016	214	RCT	Heroin-assisted treatment	24	7.34	Contingent vouchers vs. No vouchers	LDA	Yes	1

MOUD = Medications for opioid use disorder; CM = contingency management; Tx = Treatment; QA = Quality Assessment using the Effective Public Health Practice Project tool (Thomas et al., 2004); RCT = Randomized control trial; M = Methadone; B = Buprenorphine; N = Naltrexone; LDA = Longest duration of abstinence; CBT = Cognitive behavioral therapy; CNBD = Could not be determined; LAAM = levo-alpha-acetylmethadol; TOP = topiramate

[a] Scores do not include the blinding quality assessment measure. See e-Table 6 for scores including the blinding measure.

[b] Noncontingent control means that vouchers of value comparable to those provided in the Contingent voucher condition were provided to participants in the Control condition but independent of stimulant use thereby keeping material resources provided to participants comparable across conditions.

[c] This study examined two magnitudes of CM versus a control condition (no CM). Max earnings per day is the average of these two conditions. Abstinence in the condition with the higher magnitude (total max earnings = $3,480) was statistically significantly greater than the lower magnitude (total max earnings = $382) and the control condition. Abstinence was not statistically significantly different in the lower magnitude group versus control.

[d] This study examined a “brief abstinence test” with non-treatment seeking individuals.

[e] Data were used from the maintenance phase where participants were re-randomized to new study groups.

[f] Follow-up data were recorded from Defulio and Silverman (2011).[72]

[g] Aftercare = Following voucher-based CM, participants in after received $1.00 state lottery tickets for submitting negative samples 2x weekly.

© 2021 Bolívar HA et al. *JAMA Psychiatry*.

eTable 2. Studies examining abstinence from multiple substances (n=23).

Author	Year	N	Study Design	MOUD	CM duration (weeks)	Max earnings/ day ($)	Conditions Compared	Drugs targeted	Outcome	Statistically significant effect at end of Tx?	QA Rating[a]
Iguchi et al.[23]	1997	103	RCT	M	12	2.14	Contingent vouchers vs. No vouchers	Opiates, barbiturates, benzodiazepines, cocaine, tetra-hydrocannabinol, amphetamines, propoxyphene hydrochloride (Darvon)	"Percent clinically improved'	Yes	1
Piotrowski et al.[24]	1999	102	RCT	M	17	6.34	Contingent vouchers vs. No vouchers	Amphetamines, barbiturates, benzodiazepine, cocaine, heroin, tetra-hydrocannabinol, alcohol	LDA	Yes	2
Downey et al.[25]	2000	41	RCT	B	12	11.88	Contingent vouchers vs. Noncontingent vouchers[b] (yoked)	Amphetamine, barbiturates, cocaine, heroin, phencyclidine, alcohol	% 1 or more drug free urines	No	3
Dallery et al.[26]	2001	15	Within-subj	M	9	29.71	High or low magnitude vouchers vs. No voucher baseline	Opioids and cocaine	% of drug negative samples	Yes	2
Carroll et al.[27,c]	2001	127	RCT	N	12	3.34	Contingent vouchers vs. No voucher	Opioids, cocaine, benzodiazepines	Number of drug-free urines	Yes	3
Carroll et al.[28,c]	2002	55	RCT	N	12	5.10	Contingent vouchers vs. No voucher	Opioids, cocaine, benzodiazepines	Number of drug-free urines	No	2
Petry et al.[29]	2002	42	RCT	M	12	4.04	Contingent vouchers vs. No voucher	Opioids and cocaine	LDA	Yes	1
Kosten et al.[30,d]	2003a	160	RCT	B	12	8.79	Contingent vouchers +	Opioids and cocaine	Consecutive weeks abstinent	Yes	2

© 2021 Bolívar HA et al. *JAMA Psychiatry.*

							placebo or desipramine vs. Noncontingent vouchers (yoked) + placebo or desipramine				
Katz et al.[31,e]	2004	211	RCT	B	0.71	20.12	Contingent vs. Noncontingent vouchers	Opioids and cocaine	% of individuals negative	Yes	3
Schottenfeld et al.[32]	2005	162	RCT	M, B	12	11.88	Contingent vouchers + M or B vs. No vouchers + M or B	Opioids and cocaine	LDA	Yes	2
Oliveto et al.[33]	2005	140	RCT	LAAM	12	8.79	Contingent vouchers + LAAM (high or low dose) vs. Noncontingent vouchers (yoked) + LAAM (high or low dose)	Opioids and cocaine	% negative urine samples	Yes	2
Peirce et al.[34]	2006	402	RCT	M	12	4.76	Contingent prize draws vs. No vouchers	Cocaine, amphetamine, methamphetamine, and alcohol	LDA	Yes	1
Poling et al.[35]	2006	106	RCT	M	12	5.50	Contingent vouchers + buproprion or placebo vs. Noncontingent vouchers + buproprion or placebo	Opioids and cocaine	LDA	Yes	1
Knealing et al.[36]	2006	47	RCT	M	36	22.69	Contingent access to work and earn vouchers vs. No vouchers (usual care)	Opioids, cocaine, and alcohol	Rate of negative samples	No	2

© 2021 Bolívar HA et al. *JAMA Psychiatry*.

Gross et al.[37]	2006	60	RCT	B	12	3.20	Contingent vouchers vs. No vouchers	Opioids and cocaine	Continuous abstinence	No	1
Brooner et al.[38]	2007	236	RCT	M	24	19.05	Contingent vouchers with and without stepped care vs. No Vouchers with and without stepped care	Opioids, barbiturates, cocaine, alcohol, benzodiazepines	% of negative urines	Yes	2
Bickel et al.[39]	2008	135	RCT	B	23	8.18	Contingent vouchers + therapist- or computer-delivered community reinforcement vs. No vouchers	Opioids and cocaine	LDA	Yes	2
Epstein et al.[40]	2009	252	RCT	M	12	13.75	Contingent vouchers + Constant or increased M dose vs. Noncontingent vouchers + Constant or increased M dose	Opioids and cocaine	LDA	Yes	1
Chopra et al.[41]	2009	120	RCT	B	12	11.88	Computerized community reinforcement + contingent vouchers vs. No vouchers	Opioids and cocaine	LDA	No	1
Tuten et al.[42]	2012a	133	RCT	M	13	14.99	Escalating or fixed contingent vouchers vs. Noncontingent vouchers (yoked)	Opioids and cocaine	Longest number of time points negative	No	2

© 2021 Bolívar HA et al. *JAMA Psychiatry.*

Petry et al.[43]	2012	130	RCT	M	12	CNBD	Contingent prize draws vs. No draws	Alcohol and cocaine	LDA	Yes	1
Holtyn et al.[44]	2014	98	RCT	M	26	CNBD	Contingent work access vs. Noncontingent work access	Opioids and cocaine	% samples negative for cocaine & opiates	No	2
Petry et al.[45]	2015	240	RCT	M	12	8.33	Contingent prize drawings or vouchers vs. No vouchers	Alcohol and cocaine	LDA	Yes	1

MOUD = Medications for opioid use disorder; CM = contingency management; Tx = Treatment; QA = Quality Assessment using the Effective Public Health Practice Project tool (Thomas et al., 2004); RCT = Randomized control trial; M = Methadone; B = Buprenorphine; N = Naltrexone; CNBD = Could not be determined; LDA = Longest duration of abstinence; LAAM = levo-alpha-acetylmethadol.

[a] Scores do not include the blinding quality assessment measure. See e-Table 6 for scores including the blinding measure.

[b] Noncontingent control means that vouchers of value comparable to those provided in the Contingent voucher condition were provided to participants in the Control condition but independent of polysubstance use thereby keeping material resources provided to participants comparable across conditions.

[c] Carroll et al. (2001, 2002) provided vouchers on two independent tracks (abstinence from multiple substances and naltrexone adherence). This value reports max earnings possible for the abstinence track only. See Table 5 for max earnings per day for the naltrexone adherence track.

[d] Kosten et al. (2003)[73] reports follow-up from a continuation follow-up to Kosten et al. (2003)[30] in which the number of samples required to earn a voucher progressively increased. This study showed a gradual reduction in the efficacy of CM as the response requirement increased.

[e] This study examined a “brief abstinence test” with non-treatment seeking individuals.

© 2021 Bolívar HA et al. *JAMA Psychiatry.*

eTable 3. Studies examining abstinence from illicit opioids (n=11).

Author	Year	N	Study Design	MOUD	CM duration (weeks)	Max earnings/day ($)	Conditions Compared	Outcomes	Statistically significant effect at end of Tx?	QA Rating[a]
McCaul et al.[46]	1984	20	RCT	M	10	2.86	Contingent vs. Noncontingent vouchers[b]	% of samples positive	Yes	3
Silverman et al.[47]	1996b	13	Within-subj	M	12	13.75	Contingent vs. No vouchers	Mean daily % of samples positive	Yes	1
Preston et al.[48]	2000	120	RCT	M	8	9.89	Contingent vs. Noncontingent vouchers	% negative samples	Yes	1
Robles et al.[49]	2002	48	RCT	M	22	14.49	Contingent vs. Noncontingent vouchers (yoked)	LDA	Yes	1
Correia et al.[50,c]	2003	58	Within-subj	M	0.71	40.00	Contingent vouchers vs. No Vouchers	% of participants abstinent	No	2
Hser et al.[51]	2011	319	RCT	M	12	CNBD	Contingent vouchers vs. No vouchers	LDA	Yes	1
Jiang et al.[52]	2012	160	RCT	M	12	CNBD	Contingent vouchers vs. No vouchers	LDA	No	1
Chen et al.[53]	2013	246	Randomized by clinic	M	12	CNBD	Contingent vouchers vs. No vouchers	Number of negative samples	Yes	1
Ling et al.[54]	2013	202	RCT	B	16	CNBD	Contingent vouchers vs. No vouchers	LDA	No	3
Wang et al.[55]	2014	2662	Clinic-assignment	M	24	CNBD	Contingent vouchers vs. No vouchers	% urine positive	No	3
Jarvis et al.[56]	2019	84	RCT	N	24	CNBD	Contingent work access vs. Noncontingent work access	% weekly samples negative	Yes	3

© 2021 Bolívar HA et al. *JAMA Psychiatry.*

MOUD = Medications for opioid use disorder; CM = contingency management; Tx = Treatment; QA = Quality Assessment using the Effective Public Health Practice Project tool (Thomas et al., 2004); RCT = Randomized control trial; M = Methadone; B = Buprenorphine; N = Naltrexone; CNBD = Could not be determined; LDA = Longest duration of abstinence

[a] Scores do not include the blinding quality assessment measure. See e-Table 6 for scores including the blinding measure.

[b] Noncontingent control means that vouchers of value comparable to those provided in the Contingent voucher condition were provided to participants in the Control condition but independent of illicit opioid use thereby keeping material resources provided to participants comparable across conditions.

[c] This study examined a “brief abstinence test” with non-treatment seeking individuals.

© 2021 Bolívar HA et al. *JAMA Psychiatry*.

eTable 4. Studies targeting abstinence from cigarette smoking (n=5).

Author	Year	N	Study Design	MOUD	CM duration (weeks)	Maximum earnings /day ($)	Conditions Compared	Outcome	Statistically significant effect at end of Tx?	QA Rating[a]
Shoptaw et al.[57]	2002	175	RCT	M	12	5.33	Contingent vouchers + NRT patch vs. NRT patch only	% smoking-abstinent	Yes	2
Dunn et al.[58]	2008	20	RCT	M	2	25.89	Contingent vs. Noncontingent vouchers[b] (yoked)	LDA	Yes	1
Dunn et al.[59]	2010	40	RCT	M or B	2	25.89	Contingent vouchers vs. Noncontingent vouchers	% negative samples	Yes	1
Tuten et al.[60]	2012b	102	RCT	M	12	10.21	Contingent vouchers vs. Noncontingent vouchers	Mean carbon monoxide levels	Yes	2
Sigmon et al.[61]	2016	63	RCT	M or B	10	8.14	Contingent vouchers vs. Noncontingent vouchers	LDA	No	1

MOUD = Medications for opioid use disorder; CM = contingency management; Tx = Treatment; QA = Quality Assessment using the Effective Public Health Practice Project tool (Thomas et al., 2004); RCT = Randomized control trial; M = Methadone; B = Buprenorphine; NRT = Nicotine replacement therapy; LDA = Longest duration of abstinence

[a] Scores do not include the blinding quality assessment measure. See e-Table 6 for scores including the blinding measure

[b] Noncontingent control means that vouchers of value comparable to those provided in the Contingent voucher condition were provided to participants in the Control condition but independent of cigarette smoking thereby keeping material resources provided to participants comparable across conditions.

© 2021 Bolívar HA et al. *JAMA Psychiatry.*

eTable 5. Studies targeting therapy attendance or medication adherence outcomes (n=18)

Author	Year	N	Study Design	MOUD	CM duration (weeks)	Maximum earnings/day ($)	Conditions Compared	Outcome	Statistically significant effect at end of Tx?	QA Rating[a]
A. Therapy Attendance / Retention (n=11)										
Jones et al.[62]	2000	25	RCT	M	1	12.14	Contingent vouchers vs. No vouchers	Tx days attended	Yes	2
Rhodes et al. (Exp 1)[63]	2003	62	Within-Subj	M	8	CNBD	Contingent draws vs. No draws	% on-time counseling appointments attended	No	1
Rhodes et al. (Exp 2)[63]	2003	70	Within-subj	M	8	CNBD	Contingent draws vs. No draws	% on-time counseling appointments attended	No	1
Petry et al. (cont'd)[12]	2005	77	RCT	M	12	CNBD	Contingent prize draws vs. No prize draws	# group sessions attended	Yes	1
Rowan-Szal et al. (cont'd)[11]	2005	61	RCT	M	8	0.45	Contingent vouchers with or without counseling vs. No Vouchers with or without counseling	Average # individual counseling sessions attended	No	2
Hser et al. (cont'd)[51]	2011	319	RCT	M	12	CNBD	Contingent vouchers vs. No vouchers	% of subjects retained	Yes	1
Jiang et al. (cont'd)[52]	2012	160	RCT	M	12	CNBD	Contingent vouchers vs. No vouchers	% of subjects retained	No	1
Kidorf et al.[64]	2013	125	RCT	M	12	3.57	Contingent vouchers vs. No vouchers	Sessions attended	Yes	1
Chen et al. (cont’d)[53]	2013	246	Randomized by clinic	M	12	CNBD	Contingent vouchers vs. No vouchers	Treatment days attended	Yes	1

© 2021 Bolívar HA et al. *JAMA Psychiatry.*

Holtyn et al. (cont'd)[44]	2014	98	RCT	M	26	28.57	Contingent work access vs. Noncontingent work[b] access	M enrollment	No	2
Kidorf et al.[65]	2018	212	RCT	M	13	CNBD	Contingent vouchers vs. No vouchers	# counseling sessions attended	No	2
B. Medication Adherence (n=9)										
Preston et al.[66]	1999	58	RCT	N	12	13.75	Contingent vouchers vs. No vouchers or noncontingent vouchers	Maximum # N doses	Yes	1
Carroll et al. (cont'd)[27]	2001	127	RCT	N	12	3.34	Contingent vouchers vs. No voucher	# of N doses	No	3
Carroll et al. (con'td)[28]	2002	55	RCT	N	12	5.10	Contingent vouchers vs. No voucher	# of N doses	No	2
Sorensen et al.[67]	2007	66	RCT	M	12	13.96	Contingent vouchers + counseling vs. Counseling only	% adherence to HAART	Yes	1
Everly et al.[68]	2011	35	RCT	N	26	CNBD	Contingent work access vs. Noncontingent work access	% doses accepted	Yes	1
Defulio et al.[69]	2012	38	RCT	N	26	CNBD	Contingent work access vs. Noncontingent work access	% doses accepted	Yes	2
Dunn et al.[70,c]	2013	67	RCT	N	26	CNBD	Contingent work access vs. Noncontingent work access	% urines samples positive for N	Yes	2
Weaver et al.[71]	2014	210	RCT	"Opioid substitution therapy"	0.43	16.00	Escalating or fixed contingent vouchers vs. No vouchers	Completing Hepatitis B vaccination	Yes	2
Kidorf et al. (cont'd)[65]	2018	212	RCT	M	13	CNBD	Contingent vouchers vs. No vouchers	# of scheduled M doses taken	No	2

MOUD = Medications for opioid use disorder; CM = contingency management; Tx = Treatment; QA = Quality Assessment using the Effective Public Health Practice Project tool (Thomas et al., 2004); RCT = Randomized control trial; M = Methadone; B = Buprenorphine; N = Naltrexone; CNBD = Could not be determined; HAART = Highly active antiretroviral therapy

[a] Scores do not include the blinding quality assessment measure. See e-Table 6 for scores including the blinding measure.

© 2021 Bolívar HA et al. *JAMA Psychiatry.*

[b] Noncontingent control means that vouchers of value comparable to those provided in the Contingent voucher condition were provided to participants in the Control condition but independent of therapy attendance thereby keeping material resources provided to participants comparable across conditions.
[c] Follow-up data recorded from Dunn et al. (2015).[74]

© 2021 Bolívar HA et al. *JAMA Psychiatry*.

eTable 6. Detailed quality assessment data that were gathered using the Effective Public Health Practice Project tool (Thomas et al., 2004). Each study was evaluated by two or more reviewers independently assessed and rated each study and discrepancies were resolved via discussion.

Author	Year	Selection Bias	Study Design	Confounds	Blinding	Data Collection	Withdrawals/ Dropouts	Overall Without Blinding	Overall With Blinding
Table 1. Abstinence from Psychomotor Stimulants									
Blanken et al.	2016	1	1	1	3	1	1	1	2
Defulio et al.	2009	2	1	1	2	1	1	1	1
Epstein et al.	2003	1	1	1	3	1	2	1	2
Festinger et al.	2014	2	1	1	3	1	3	2	3
Katz et al.	2002	3	2	1	3	1	1	2	3
Kennedy et al.	2013	2	1	1	2	1	2	1	1
Kirby et al.	2013	2	1	1	3	1	2	1	2
Petry et al.	2005	1	1	1	3	1	1	1	1
Petry et al.	2007	1	1	3	3	1	1	1	2
Preston et al.	2001	2	1	3	3	1	1	2	3
Rawson et al.	2002	2	2	1	2	1	1	1	2
Robles et al.	2000	2	2	1	1	1	2	2	2
Rowan-Szal et al.	2005	1	1	1	3	1	3	2	3
Sigmon et al.	2004	1	1	1	1	1	2	1	1
Silverman et al.	1996a	1	1	1	3	1	1	1	2
Silverman et al.	1998	1	2	3	3	1	3	3	3
Silverman et al.	1999	1	2	1	3	1	2	1	2
Silverman et al.	2004	1	1	1	3	1	2	1	2
Silverman et al.	2007	3	1	1	2	1	1	1	2
Umbricht et al.	2014	1	1	1	1	1	2	1	1

© 2021 Bolívar HA et al. *JAMA Psychiatry.*

Vandrey et al.	2007	3	2	1	3	1	1	2	3
Winstanley et al.	2011	1	1	1	2	1	3	2	2
Table 2. Abstinence from Polydrug Use									
Bickel et al.	2008	1	1	1	3	1	3	2	3
Brooner et al.	2007	2	2	1	3	1	3	2	3
Carroll et al.	2001	3	1	1	3	1	3	3	3
Carroll et al.	2002	2	1	1	3	1	3	2	3
Chopra et al.	2009	2	1	1	3	1	2	1	2
Dallery et al.	2001	3	2	1	3	1	2	2	3
Downey et al.	2000	2	1	3	3	1	3	3	3
Epstein et al.	2009	2	1	1	2	1	2	1	2
Gross et al.	2006	2	1	1	3	1	2	1	2
Holtyn et al.	2014	2	1	3	3	1	1	2	3
Iguchi et al.	1997	2	1	1	3	1	2	1	2
Katz et al.	2004	3	1	2	3	1	3	3	3
Knealing et al.	2006	2	1	1	3	1	3	2	3
Kosten et al.	2003a	2	1	1	1	1	3	2	2
Oliveto et al.	2005	2	1	1	1	1	3	2	2
Peirce et al.	2006	1	1	1	3	1	1	1	1
Peles et al.	2017	2	2	3	3	3	3	3	3
Petry et al.	2002	1	1	3	3	1	1	1	2
Petry et al.	2012	2	1	1	3	1	2	1	2
Petry et al.	2015	2	1	1	3	1	1	1	2
Piotrowski et al.	1999	2	3	1	2	1	2	2	2
Poling et al.	2006	1	1	1	3	1	2	1	2
Schottenfeld et al.	2005	1	1	1	3	1	3	2	3

© 2021 Bolívar HA et al. *JAMA Psychiatry*.

Tuten et al.	2012a	3	2	1	3	1	1	2	2
Table 3. Abstinence from Illicit Opioids									
Chen et al.	2013	1	1	1	3	1	2	1	2
Correia et al.	2003	2	3	1	3	1	2	2	3
Hser et al.	2011	2	1	1	3	1	2	1	2
Jarvis et al.	2017	1	1	1	3	3	3	3	3
Jiang et al.	2012	2	1	1	2	1	1	1	1
Ling et al.	2013	2	1	3	3	1	3	3	3
McCaul et al.	1984	2	1	3	3	1	3	3	3
Preston et al.	2000	2	2	1	2	1	1	1	2
Robles et al.	2002	1	1	1	3	1	2	1	2
Silverman et al.	1996b	1	2	1	3	1	1	1	2
Wang et al.	2014	1	3	3	3	1	3	3	3
Table 4. Abstinence from Cigarette Smoking									
Dunn et al.	2008	2	1	1	3	1	2	1	2
Dunn et al.	2010	1	1	1	3	1	2	1	2
Shoptaw et al.	2002	1	1	1	3	1	3	2	3
Sigmon et al.	2016	2	1	1	3	1	2	1	2
Tuten et al.	2012b	2	1	1	3	1	3	2	2
Table 5. Therapy Attendance and Medication Adherence									
5A.Therapy Attendance									
Jones et al.	2000	2	1	1	3	1	3	2	2
Kidorf et al.	2013	1	1	1	3	1	1	1	1
Kidorf et al.	2018	1	1	1	2	1	3	2	2
Rhodes et al.	2003	2	2	1	2	1	2	1	1
5B. Medication Adherence									

© 2021 Bolívar HA et al. *JAMA Psychiatry.*

Defulio et al.	2012	2	1	1	3	1	3	2	3
Dunn et al.	2013	2	1	1	3	1	3	2	3
Everly et al.	2011	1	1	1	3	1	2	1	2
Preston et al.	1999	1	2	1	3	1	2	1	2
Sorensen et al.	2007	1	1	1	3	1	1	1	2
Weaver et al.	2014	2	1	1	3	1	3	2	3

© 2021 Bolívar HA et al. *JAMA Psychiatry*.

eTable 7. Studies excluded at full-text review with reasons for exclusion.

**Study**	**Reason for exclusion**
Acevedo et al. 2018	Did not test CM among participants on MOUD
Ainscough et al. 2017a	Not an original study, did not use a prospective design, did not include a comparison condition, did not use a design that isolates CM effect, did not test CM among participants on MOUD, included <10 participants
Ainscough et al. 2017b	Did not involve monetary-based CM, not an original study, did not use a prospective design, did not include a comparison condition, did not use a design that isolates CM effect, did not test CM among participants on MOUD
Alessi & Petry 2014	Did not test CM among participants on MOUD
Alessi et al. 2007	Did not test CM among participants on MOUD
Alessi et al. 2008	Did not test CM among participants on MOUD
Alessi et al. 2020	Did not use a design that isolates CM effect, did not test CM among participants on MOUD
Alessi et al. 2017	Did not test CM among participants on MOUD
Barnett et al. 2017	Did not test CM among participants on MOUD
Barnett et al. 2009	Not an original study, did not use a prospective design, included <10 participants
Barry et al. 2009	Not an original study, included <10 participants
Barry et al. 2008	Not an original study
Bickel et al. 1997	Did not use a design that isolates CM effect
Bickel et al. 1988	Did not involve monetary-based CM, included <10 participants
Bigelow et al. 1980	Did not involve monetary-based CM
Branson et al. 2012	Did not use a prospective design, did not test CM among participants on MOUD, included <10 participants
Brewer & Hagan 2009	Did not involve monetary-based CM, not an original study, did not include a comparison condition, did not use a design that isolates CM effect, did not test CM among participants on MOUD, included <10 participants
Bride & Humble 2008	Did not use a prospective design, did not test CM among participants on MOUD
Brolin et al. 2017	Did not test CM among participants on MOUD
Brooner et al. 1998	Did not involve monetary-based CM, did not use a design that isolates CM effect
Brooner et al. 2004	Did not involve monetary-based CM
Budney et al. 1991	Did not include a comparison condition, did not use a design that isolates CM effect, did not test CM among participants on MOUD, included <10 participants
Businelle et al. 2009	Not an original study, did not use a prospective design, did not test CM among participants on MOUD
Campbell et al. 2012	Not an original study, did not use a prospective design, did not include a comparison condition, did not use a design that isolates CM effect, did not test CM among participants on MOUD, included <10 participants

© 2021 Bolívar HA et al. *JAMA Psychiatry.*

Carpenedo et al. 2010	Did not use a design that isolates CM effect
Carpenter et al. 2009	Not an original study, did not use a prospective design, did not use a design that isolates CM effect, included <10 participants
Carroll et al. 2016	Did not test CM among participants on MOUD
Carroll & Weiss 2017	Did not involve monetary-based CM, not an original study, did not use a prospective design, did not include a comparison condition, did not use a design that isolates CM effect, did not test CM among participants on MOUD
Christensen et al. 2014	Did not use a design that isolates CM effect
Church et al. 2001	Did not include a comparison condition, did not use a design that isolates CM effect
Chutuape et al. 1999	Included <10 participants
Chutuape et al. 2001	Did not test CM among participants on MOUD
Correia et al. 2005	Did not use a design that isolates CM effect
Corrigan & Bogner 2007	Did not test CM among participants on MOUD
Davis et al. 2016	Did not involve monetary-based CM, not an original study, did not use a prospective design, did not include a comparison condition, did not use a design that isolates CM effect, did not test CM among participants on MOUD
Day et al. 2016	Not an original study, did not test CM among participants on MOUD
De Crescenzo et al. 2018	Did not involve monetary-based CM, not an original study, did not use a prospective design, did not include a comparison condition, did not use a design that isolates CM effect, did not test CM among participants on MOUD
De Giorgi et al. 2018	Did not involve monetary-based CM, not an original study, did not use a prospective design, did not include a comparison condition, did not use a design that isolates CM effect, did not test CM among participants on MOUD
Drummond et al. 2014	Did not test CM among participants on MOUD
Dugosh et al. 2016	Did not involve monetary-based CM, not an original study, did not use a prospective design, did not include a comparison condition, did not use a design that isolates CM effect, did not test CM among participants on MOUD
Dunn et al. 2014	Did not test CM among participants on MOUD
Dunn et al. 2011	Not an original study, included <10 participants
Dunn et al. 2015	Not an original study
Elk et al. 1993	Did not involve monetary-based CM, included <10 participants
Ferrell et al. 2006	Not published in peer-reviewed journal (poster abstract)
Festinger et al. 2005	Did not use a design that isolates CM effect, did not test CM among participants on MOUD
Festinger et al. 2008	Did not use a design that isolates CM effect, did not test CM among participants on MOUD
FitzGerald et al. 1999	Did not test CM among participants on MOUD
Fitzsimons et al. 2015	Did not test CM among participants on MOUD
Forster et al. 2019	Did not involve monetary-based CM, not an original study, did not use a prospective design, did not include a comparison

	condition, did not use a design that isolates CM effect, did not test CM among participants on MOUD
Getty et al. 2019	Did not involve monetary-based CM, not an original study, did not use a prospective design, did not include a comparison condition, did not use a design that isolates CM effect, did not test CM among participants on MOUD
Ghitza et al. 2008	Not an original study, included <10 participants
Gonzales-Nolas et al. 2019	Not published in peer-reviewed journal (poster abstract)
Gonzalez et al. 2003	Not an original study
Griffith et al. 2000	Did not involve monetary-based CM, not an original study, did not use a prospective design, did not include a comparison condition, did not use a design that isolates CM effect, did not test CM among participants on MOUD
Gruber et al. 2008	Did not involve monetary-based CM, did not use a design that isolates CM effect
Hall et al. 2017	Did not test CM among participants on MOUD
Hand et al. 2017	Did not involve monetary-based CM, not an original study, did not use a prospective design, did not include a comparison condition, did not use a design that isolates CM effect, did not test CM among participants on MOUD
Hays 2009	Did not involve monetary-based CM, not an original study, did not use a prospective design, did not include a comparison condition, did not use a design that isolates CM effect, did not test CM among participants on MOUD
Heil et al. 2016	Did not use a design that isolates CM effect
Herrmann et al. 2017	Did not involve monetary-based CM, not an original study, did not use a prospective design, did not include a comparison condition, did not use a design that isolates CM effect, did not test CM among participants on MOUD
Higgins et al. 1994	Did not test CM among participants on MOUD
Higgins et al. 1991	did not test CM among participants on MOUD
Himelhoch et al. 2017	Did not involve monetary-based CM, did not use a design that isolates CM effect, did not test CM among participants on MOUD, included <10 participants
Holtyn et al. 2014	Not an original study
Jarvis et al. 2017	Not an original study
Jones et al. 2001	Did not use a design that isolates CM effect
Katz et al. 2002	Did not test CM among participants on MOUD
Kelly et al. 2014	Did not include a comparison condition, did not use a design that isolates CM effect, did not test CM among participants on MOUD
Kidorf & Stitzer 1993	Did not involve monetary-based CM, did not test CM among participants on MOUD
Kidorf & Stitzer 1996	Did not involve monetary-based CM, included <10 participants
Kidorf et al. 1997	Did not involve monetary-based CM, did not include a comparison condition, did not use a design that isolates CM effect
Kiluk et al. 2017	Did not test CM among participants on MOUD

© 2021 Bolívar HA et al. *JAMA Psychiatry.*

Kirby et al. 2008	Did not use a design that isolates CM effect
Kosten et al. 2003	Not an original study
Kropp et al. 2017	Did not include a comparison condition
Lee et al. 2018	Did not test CM among participants on MOUD
Lussier et al. 2006	Did not involve monetary-based CM, not an original study, did not use a prospective design, did not include a comparison condition, did not use a design that isolates CM effect, did not test CM among participants on MOUD
Marino et al. 2019	Not an original study, did not test CM among participants on MOUD
Marsden et al. 2019	Did not use a design that isolates CM effect
McKay et al. 2010	Did not test CM among participants on MOUD
McPherson et al. 2018	Did not involve monetary-based CM, not an original study, did not use a prospective design, did not include a comparison condition, did not use a design that isolates CM effect, did not test CM among participants on MOUD
Messina et al. 2003	Not an original study
Metsch et al. 2016	Did not test CM among participants on MOUD
Miguel et al. 2016	Did not test CM among participants on MOUD
Murphy et al. 2018	Not an original study, did not test CM among participants on MOUD
NCT00000311. 1999	Not published in peer-reviewed journal
NCT00249535. 2005	Not published in peer-reviewed journal
NCT00249522. 2005	Not published in peer-reviewed journal
NCT00878852. 2009	Not published in peer-reviewed journal
NCT00838981. 2009	Not published in peer-reviewed journal
NCT01204879. 2010	Not published in peer-reviewed journal
Neufeld et al. 2008	Not an original study
Norton et al. 2019	Did not test CM among participants on MOUD
Olmstead & Petry 2009	Not an original study, did not use a prospective design, did not test CM among participants on MOUD, included <10 participants
Peles et al. 2017	Did not use a design that isolates CM effect
Petry et al. 2012	Did not test CM among participants on MOUD
Petry et al. 2006	Did not test CM among participants on MOUD
Petry et al. 2018	Did not test CM among participants on MOUD
Petry et al. 2010	Did not test CM among participants on MOUD
Petry, Alessi, et al. 2005	Did not test CM among participants on MOUD
Petry, Peirce, et al. 2005	Did not test CM among participants on MOUD
Petry & Carroll 2013	Not an original study, did not test CM among participants on MOUD
Petry et al. 2011	Did not test CM among participants on MOUD
Prendergast et al. 2006	Not an original study, did not use a prospective design, did not include a comparison condition, did not use a design that

© 2021 Bolívar HA et al. *JAMA Psychiatry.*

	isolates CM effect, did not test CM among participants on MOUD, included <10 participants
Preston et al. 2008	Did not use a design that isolates CM effect
Rash et al. 2017	Not an original study, did not test CM among participants on MOUD
Rawson et al. 2006	Did not test CM among participants on MOUD
Rogers et al. 2008	Not an original study
Rohsenow et al. 2015	Did not test CM among participants on MOUD
Rohsenow et al. 2017	Did not test CM among participants on MOUD
Rosen et al. 2007	Did not test CM among participants on MOUD
Rothenberg et al. 2002	Did not include a comparison, did not use a design that isolates CM effect
Rowanszal et al. 1994	Did not use a design that isolates CM effect
Sayegh et al. 2017	Did not involve monetary-based CM, not an original study, did not use a prospective design, did not include a comparison condition, did not use a design that isolates CM effect, did not test CM among participants on MOUD
Schmitz et al. 1995	Did not test CM among participants on MOUD, included <10 participants
Schroeder et al. 2006	Not an original study
Shoptaw et al. 1996	Did not include a comparison condition, did not use a design that isolates CM effect
Sigmon & Stitzer 2005	Did not include a comparison condition, did not use a design that isolates CM effect
Silverman et al. 1996	included <10 participants
Stanger et al. 2011	Did not test CM among participants on MOUD
Stitzer et al. 1984	Did not involve monetary-based CM, not published in peer-reviewed journal, not an original study, did not use a prospective design, did not include a comparison condition, did not use a design that isolates CM effect, did not test CM among participants on MOUD, included <10 participants
Stitzer et al. 2018	Not an original study
Stitzer et al. 1993	Did not involve monetary-based CM, not published in peer-reviewed journal
Stitzer et al. 2017	Not an original study, did not use a prospective design, did not include a comparison condition, did not use a design that isolates CM effect, did not test CM among participants on MOUD, included <10 participants
Subramaniam et al. 2018	Not an original study, did not test CM among participants on MOUD
Svikis et al. 1997	Did not test CM among participants on MOUD
Svikis et al. 2007	Not an original study, did not test CM among participants on MOUD
Tardelli et al. 2018	Did not involve monetary-based CM, not an original study, did not use a prospective design, did not include a comparison condition, did not use a design that isolates CM effect, did not test CM among participants on MOUD

© 2021 Bolívar HA et al. *JAMA Psychiatry.*

Thurgood et al. 2016	Did not involve monetary-based CM, not an original study, did not use a prospective design, did not include a comparison condition, did not use a design that isolates CM effect, did not test CM among participants on MOUD
Topp et al. 2013	Did not test CM among participants on MOUD
Tuten et al. 2012	Did not test CM among participants on MOUD
Van Horn et al. 2011	Not an original study, did not test CM among participants on MOUD, included <10 participants
Vanderplasschen 2008	Did not involve monetary-based CM, not published in peer-reviewed journal, not an original study, did not use a prospective design, did not include a comparison condition, did not use a design that isolates CM effect, did not test CM among participants on MOUD, included <10 participants
Versek et al. 2010	Not an original study, did not use a design that isolates CM effect
Villano et al. 2002	Did not use a design that isolates CM effect
Weinstock et al. 2010	Not an original study
Winklbaur-Hausknost et al. 2013	Not an original study, did not test CM among participants on MOUD
Wong et al. 2004	Included <10 participants

MOUD = Medications for opioid use disorder; CM = contingency management.

© 2021 Bolívar HA et al. *JAMA Psychiatry.*

eTable 8. Moderator analysis results

Moderator type	Result
Includes All Studies in Meta-analysis	
Sample size	Q = 2.26, p = 0.13
Includes Studies Targeting Abstinence[a]	
Mean Daily Earnings[c,d]	Q = 5.67, p = 0.02
CM Duration[d]	Q = 4.56, p = 0.10
Quality Score	Q = 0.03, p = 0.99
Includes Studies Targeting Treatment Attendance and Medication Adherence[b]	
Mean Daily Earnings[c,d]	Q = 4.82, p = 0.03
CM Duration[d]	Q = 0.20, p = 0.91
Quality Score	Q = 1.79, p = 0.41

CM = contingency management.

[a] All studies depicted in eFigure 1.

[b] All studies depicted in eFigure 2.

[c] Indicates a significant positive association between maximum daily earnings and effect size.

[d] Studies examining "brief abstinence tests"[4,6,31] were omitted from moderator analyses of mean daily earnings and CM duration because they included non-treatment seeking individuals.

© 2021 Bolívar HA et al. *JAMA Psychiatry.*

Study name	Statistics for each study: Cohen's d	Lower limit	Upper limit	Weight: Relative weight
Bickel et al. 2008	0.377	-0.111	0.864	2.04
Blanken et al. 2016	0.484	0.212	0.756	2.78
Brooner et al. 2007	0.473	0.107	0.839	2.45
Carroll et al. 2001	0.060	-0.384	0.504	2.19
Carroll et al. 2002	0.520	-0.054	1.094	1.78
Chen et al. 2013	0.571	0.316	0.826	2.83
Correia et al. 2003	0.714	0.183	1.245	1.91
DeFulio et al. 2009	0.726	0.158	1.294	1.80
Downey et al. 2000	0.243	-0.372	0.858	1.67
Dunn et al. 2010	1.025	0.366	1.684	1.55
Epstein et al. 2003	0.758	0.343	1.172	2.28
Epstein et al. 2009	0.506	0.083	0.929	2.26
Festinger et al. 2014	0.557	0.231	0.883	2.59
Gross et al. 2006	-0.338	-0.962	0.286	1.64
Hser et al. 2011	0.227	0.007	0.447	2.94
Iguchi et al. 1997	0.337	-0.146	0.819	2.06
Jarvis et al. 2019	0.383	-0.188	0.954	1.79
Jiang et al. 2012	0.271	-0.040	0.582	2.64
Katz et al. 2002	0.614	0.165	1.063	2.17
Katz et al. 2004	0.414	0.141	0.687	2.77
Kirby et al. 2013	0.575	0.224	0.926	2.50
Kosten et al. 2003	0.204	-0.198	0.606	2.33
Ling et al. 2013	0.276	-0.118	0.670	2.36
McCaul et al. 1984	0.944	0.020	1.868	1.03
Oliveto et al. 2005	2.058	1.479	2.637	1.76
Peirce et al. 2006	0.513	0.311	0.715	2.99
Petry et al. 2005	0.470	0.017	0.923	2.16
Petry et al. 2007	0.568	-0.028	1.164	1.72
Petry et al. 2012	0.780	0.422	1.138	2.48
Petry et al. 2015	0.504	0.137	0.871	2.45
Petry Martin et al. 2002	0.693	0.068	1.318	1.64
Piotrowski et al. 1999	0.563	0.167	0.959	2.35
Preston et al. 2000	2.065	1.422	2.708	1.59
Preston et al. 2001	0.442	-0.002	0.886	2.19
Rawson et al. 2002	0.506	-0.012	1.024	1.95
Robles et al. 2002	0.431	-0.143	1.005	1.78
Rowan-Szal et al. 2005	0.539	0.028	1.050	1.97
Schottenfeld et al. 2005	0.318	0.008	0.628	2.65
Sigmon et al. 2016	0.461	-0.039	0.961	2.00
Silverman et al. 1996	1.186	0.487	1.885	1.46
Silverman et al. 1998	5.213	3.883	6.543	0.59
Silverman et al. 1999	0.929	0.294	1.564	1.61
Silverman et al. 2004	0.975	0.400	1.550	1.78
Silverman et al. 2007	0.890	0.341	1.439	1.85
Tuten et al. 2012a	0.166	-0.240	0.571	2.32
Tuten et al. 2012b	0.950	0.456	1.444	2.02
Umbricht et al. 2014	0.118	-0.308	0.544	2.25
Winstanley et al. 2011	0.465	-0.005	0.935	2.10
**Total (95% C.I.)**	**0.580**	**0.467**	**0.693**	**100.00**

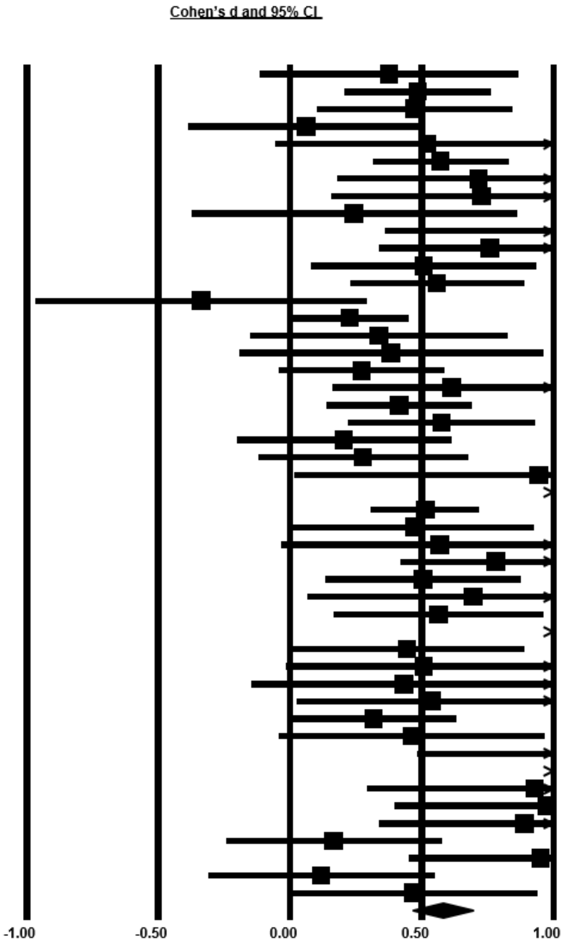

eFigure 1. Forest plot demonstrating individual and overall Cohen's *d* for all studies that targeted abstinence from substances as outcomes. These studies are in the forest plots in the main text, Figures 2-4.

© 2021 Bolívar HA et al. *JAMA Psychiatry.*

Study name	Statistics for each study			Weight
	Cohen's d	Lower limit	Upper limit	Relative weight
Carroll et al. 2001	0.045	-0.399	0.489	5.85
Carroll et al. 2002	0.520	-0.054	1.094	5.05
Chen et al. 2013	0.348	0.096	0.600	6.94
DeFulio et al. 2012	0.988	0.314	1.662	4.48
Dunn et al. 2013	1.191	0.671	1.711	5.38
Everly et al. 2011	0.958	0.258	1.658	4.34
Hser et al. 2011	0.404	0.179	0.629	7.07
Jiang et al. 2012	0.061	-0.249	0.371	6.64
Jones et al. 2000	0.729	-0.081	1.539	3.79
Kidorf et al. 2013	1.047	0.673	1.421	6.27
Kidorf et al. 2018	-0.110	-0.440	0.221	6.52
Petry et al. 2005	0.967	0.495	1.439	5.67
Preston et al. 1999	0.922	0.253	1.591	4.51
Rhodes et al. 2003 exp 1	0.173	-0.382	0.728	5.16
Rhodes et al. 2003 exp 2	0.386	-0.163	0.935	5.21
Rowan-Szal et al. 2005	0.495	-0.015	1.005	5.44
Sorenson et al. 2007	1.084	0.567	1.601	5.40
Weaver et al. 2014	1.415	1.041	1.788	6.27
**Total (95% C.I.)**	**0.621**	**0.399**	**0.844**	**100.00**

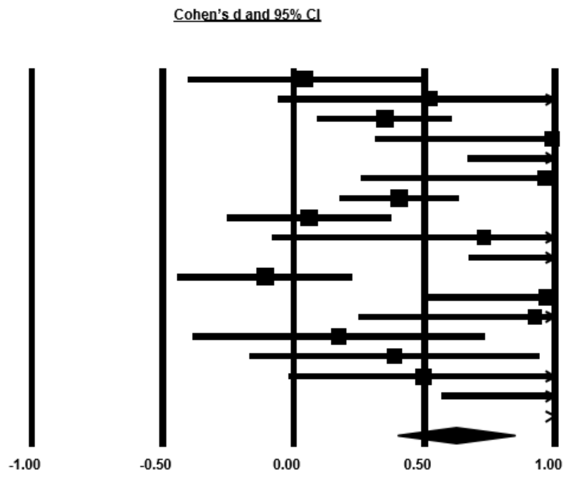

eFigure 2. Forest plot demonstrating individual and overall Cohen's *d* for all studies that targeted treatment adherence (i.e., therapy attendance or medication adherence) as outcomes. These studies are in the forest plots in the main text, Figure 5.

© 2021 Bolívar HA et al. *JAMA Psychiatry.*

Study name	Statistics for each study			Weight
	Cohen's d	Lower limit	Upper limit	Relative weight
DeFulio et al. 2011	-0.023	-0.573	0.527	9.51
Dunn et al. 2015	-0.295	-0.777	0.187	11.75
Jiang et al. 2012	-0.080	-0.390	0.230	21.67
Ling et al. 2013	0.063	-0.329	0.455	15.96
Petry et al. 2012	-0.147	-0.493	0.199	18.92
Rawson et al. 2002	0.366	-0.145	0.877	10.71
Sorenson et al. 2007	0.485	-0.005	0.975	11.46
**Total (95% C.I.)**	**0.023**	**-0.164**	**0.210**	**100.00**

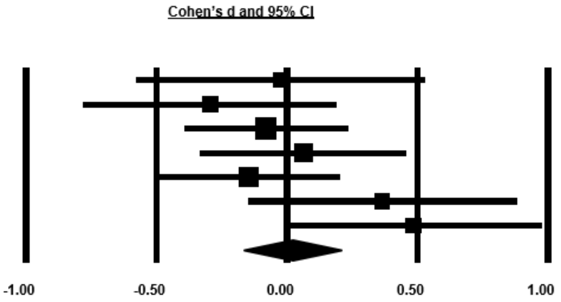

eFigure 3. Forest plot demonstrating the effect size for the seven studies for which we could calculate follow-up effect sizes.

© 2021 Bolívar HA et al. *JAMA Psychiatry*.

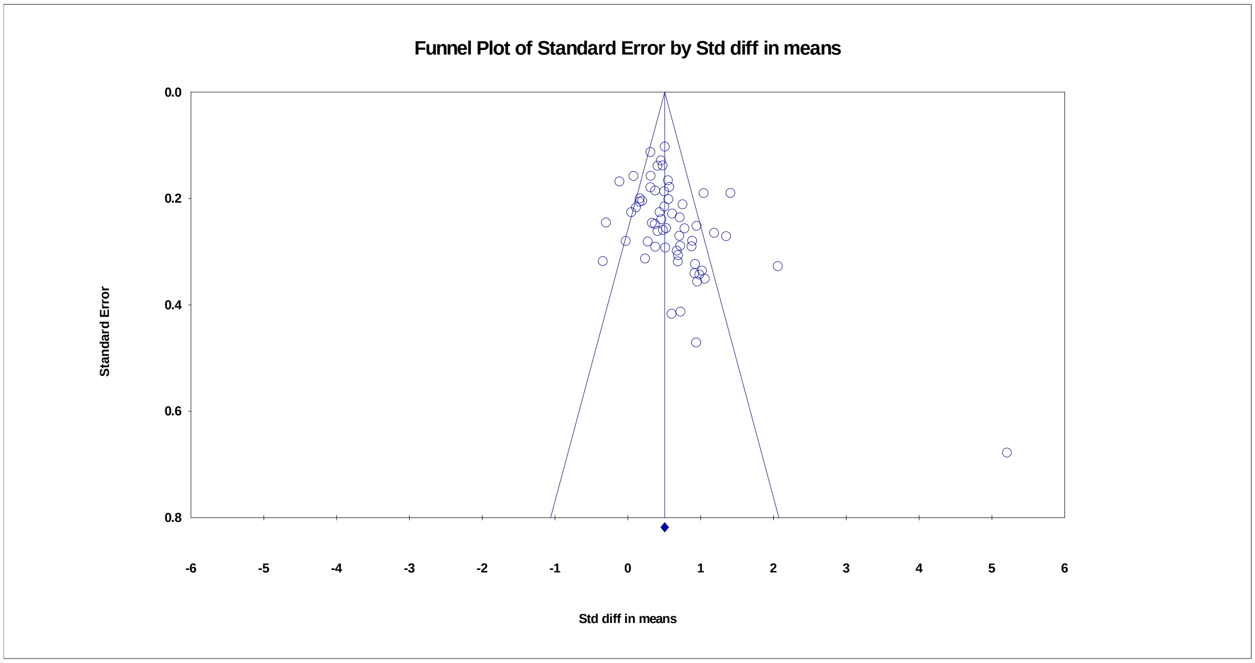

eFigure 4. Funnel plot assessing for publication bias among studies included in the meta-analysis.

© 2021 Bolívar HA et al. *JAMA Psychiatry*.

eReferences

1. Silverman K, Higgins ST, Brooner RK, et al. Sustained cocaine abstinence in methadone maintenance patients through voucher-based reinforcement therapy. *Arch Gen Psychiatry*. 1996a;53(5):409-415. doi:10.1001/archpsyc.1996.01830050045007
2. Silverman K, Wong CJ, Umbricht-Schneiter A, Montoya ID, Schuster CR, Preston KL. Broad beneficial effects of cocaine abstinence reinforcement among methadone patients. *J Consult Clin Psychol*. 1998;66(5):811-824. doi:10.1037//0022-006x.66.5.811
3. Silverman K, Chutuape MA, Bigelow GE, Stitzer ML. Voucher-based reinforcement of cocaine abstinence in treatment-resistant methadone patients: effects of reinforcement magnitude. *Psychopharmacology (Berl)*. 1999;146(2):128-138. doi:10.1007/s002130051098
4. Robles E, Silverman K, Preston KL, et al. The brief abstinence test: voucher-based reinforcement of cocaine abstinence. *Drug Alcohol Depend*. 2000;58(1-2):205-212. doi:10.1016/s0376-8716(99)00090-3
5. Preston KL, Umbricht A, Wong CJ, Epstein DH. Shaping cocaine abstinence by successive approximation. *J Consult Clin Psychol*. 2001;69(4):643-654. doi:10.1037//0022-006x.69.4.643

© 2021 Bolívar HA et al. *JAMA Psychiatry*.

6. Katz EC, Chutuape MA, Jones HE, Stitzer ML. Voucher reinforcement for heroin and cocaine abstinence in an outpatient drug-free program. *Exp Clin Psychopharmacol*. 2002;10(2):136-143. doi:10.1037//1064-1297.10.2.136
7. Rawson RA, Huber A, McCann M, et al. A comparison of contingency management and cognitive-behavioral approaches during methadone maintenance treatment for cocaine dependence. *Arch Gen Psychiatry*. 2002;59(9):817-824. doi:10.1001/archpsyc.59.9.817
8. Epstein DH, Hawkins WE, Covi L, Umbricht A, Preston KL. Cognitive-behavioral therapy plus contingency management for cocaine use: findings during treatment and across 12-month follow-up. *Psychol Addict Behav*. 2003;17(1):73-82. doi:10.1037/0893-164x.17.1.73
9. Sigmon SC, Correia CJ, Stitzer ML. Cocaine abstinence during methadone maintenance: effects of repeated brief exposure to voucher-based reinforcement. *Exp Clin Psychopharmacol*. 2004;12(4):269-275. doi:10.1037/1064-1297.12.4.269
10. Silverman K, Robles E, Mudric T, Bigelow GE, Stitzer ML. A randomized trial of long-term reinforcement of cocaine abstinence in methadone-maintained patients who inject drugs. *J Consult Clin Psychol*. 2004;72(5):839-854. doi:10.1037/0022-006X.72.5.839

© 2021 Bolívar HA et al. *JAMA Psychiatry*.

11. Rowan-Szal GA, Bartholomew NG, Chatham LR, Simpson DD. A combined cognitive and behavioral intervention for cocaine-using methadone clients. *J Psychoactive Drugs*. 2005;37(1):75-84. doi:10.1080/02791072.2005.10399750

12. Petry NM, Peirce JM, Stitzer ML, et al. Effect of prize-based incentives on outcomes in stimulant abusers in outpatient psychosocial treatment programs: a national drug abuse treatment clinical trials network study. *Arch Gen Psychiatry*. 2005;62(10):1148-1156. doi:10.1001/archpsyc.62.10.1148

13. Silverman K, Wong CJ, Needham M, et al. A randomized trial of employment-based reinforcement of cocaine abstinence in injection drug users. *J Appl Behav Anal*. 2007;40(3):387-410. doi:10.1901/jaba.2007.40-387

14. Petry NM, Alessi SM, Hanson T, Sierra S. Randomized trial of contingent prizes versus vouchers in cocaine-using methadone patients. *J Consult Clin Psychol*. 2007;75(6):983-991. doi:10.1037/0022-006X.75.6.983

15. Vandrey R, Bigelow GE, Stitzer ML. Contingency management in cocaine abusers: a dose-effect comparison of goods-based versus cash-based incentives. *Exp Clin Psychopharmacol*. 2007;15(4):338-343. doi:10.1037/1064-1297.15.4.338

16. DeFulio A, Donlin WD, Wong CJ, Silverman K. Employment-based abstinence reinforcement as a maintenance intervention for the treatment of cocaine dependence: a randomized controlled trial. *Addiction*. 2009;104(9):1530-1538. doi:10.1111/j.1360-0443.2009.02657.x

© 2021 Bolívar HA et al. *JAMA Psychiatry*.

17. Winstanley EL, Bigelow GE, Silverman K, Johnson RE, Strain EC. A randomized controlled trial of fluoxetine in the treatment of cocaine dependence among methadone-maintained patients. *J Subst Abuse Treat*. 2011;40(3):255-264. doi:10.1016/j.jsat.2010.11.010
18. Kirby KC, Carpenedo CM, Dugosh KL, et al. Randomized clinical trial examining duration of voucher-based reinforcement therapy for cocaine abstinence. *Drug Alcohol Depend*. 2013;132(3):639-645. doi:10.1016/j.drugalcdep.2013.04.015
19. Kennedy AP, Phillips KA, Epstein DH, Reamer DA, Schmittner J, Preston KL. A randomized investigation of methadone doses at or over 100 mg/day, combined with contingency management. *Drug Alcohol Depend*. 2013;130(1-3):77-84. doi:10.1016/j.drugalcdep.2012.10.025
20. Festinger DS, Dugosh KL, Kirby KC, Seymour BL. Contingency management for cocaine treatment: cash vs. vouchers. *J Subst Abuse Treat*. 2014;47(2):168-174. doi:10.1016/j.jsat.2014.03.001
21. Umbricht A, DeFulio A, Winstanley EL, et al. Topiramate for cocaine dependence during methadone maintenance treatment: a randomized controlled trial. *Drug Alcohol Depend*. 2014;140:92-100. doi:10.1016/j.drugalcdep.2014.03.033
22. Blanken P, Hendriks VM, Huijsman IA, van Ree JM, van den Brink W. Efficacy of cocaine contingency management in heroin-assisted treatment: Results of a

© 2021 Bolívar HA et al. *JAMA Psychiatry*.

randomized controlled trial. *Drug Alcohol Depend*. 2016;164:55-63. doi:10.1016/j.drugalcdep.2016.04.018

23. Iguchi MY, Belding MA, Morral AR, Lamb RJ, Husband SD. Reinforcing operants other than abstinence in drug abuse treatment: an effective alternative for reducing drug use. *J Consult Clin Psychol*. 1997;65(3):421-428. doi:10.1037//0022-006x.65.3.421
24. Piotrowski NA, Tusel DJ, Sees KL, et al. Contingency contracting with monetary reinforcers for abstinence from multiple drugs in a methadone program. *Exp Clin Psychopharmacol*. 1999;7(4):399-411. doi:10.1037//1064-1297.7.4.399
25. Downey KK, Helmus TC, Schuster CR. Treatment of heroin-dependent poly-drug abusers with contingency management and buprenorphine maintenance. *Exp Clin Psychopharmacol*. 2000;8(2):176-184. doi:10.1037//1064-1297.8.2.176
26. Dallery J, Silverman K, Chutuape MA, Bigelow GE, Stitzer ML. Voucher-based reinforcement of opiate plus cocaine abstinence in treatment-resistant methadone patients: effects of reinforcer magnitude. *Exp Clin Psychopharmacol*. 2001;9(3):317-325. doi:10.1037//1064-1297.9.3.317
27. Carroll KM, Ball SA, Nich C, et al. Targeting behavioral therapies to enhance naltrexone treatment of opioid dependence: efficacy of contingency management and significant other involvement. *Arch Gen Psychiatry*. 2001;58(8):755-761. doi:10.1001/archpsyc.58.8.755

© 2021 Bolívar HA et al. *JAMA Psychiatry*.

28. Carroll KM, Sinha R, Nich C, Babuscio T, Rounsaville BJ. Contingency management to enhance naltrexone treatment of opioid dependence: a randomized clinical trial of reinforcement magnitude. *Exp Clin Psychopharmacol*. 2002;10(1):54-63. doi:10.1037//1064-1297.10.1.54

29. Petry NM, Martin B. Low-cost contingency management for treating cocaine- and opioid-abusing methadone patients. *J Consult Clin Psychol*. 2002;70(2):398-405. doi:10.1037//0022-006x.70.2.398

30. Kosten T, Oliveto A, Feingold A, et al. Desipramine and contingency management for cocaine and opiate dependence in buprenorphine maintained patients. *Drug Alcohol Depend*. 2003;70(3):315-325. doi:10.1016/s0376-8716(03)00032-2

31. Katz EC, Chutuape MA, Jones H, Jasinski D, Fingerhood M, Stitzer M. Abstinence incentive effects in a short-term outpatient detoxification program. *Exp Clin Psychopharmacol*. 2004;12(4):262-268. doi:10.1037/1064-1297.12.4.262

32. Schottenfeld RS, Chawarski MC, Pakes JR, Pantalon MV, Carroll KM, Kosten TR. Methadone versus buprenorphine with contingency management or performance feedback for cocaine and opioid dependence. *Am J Psychiatry*. 2005;162(2):340-349. doi:10.1176/appi.ajp.162.2.340

33. Oliveto A, Poling J, Sevarino KA, et al. Efficacy of dose and contingency management procedures in LAAM-maintained cocaine-dependent patients. *Drug Alcohol Depend*. 2005;79(2):157-165. doi:10.1016/j.drugalcdep.2005.01.007

© 2021 Bolívar HA et al. *JAMA Psychiatry*.

34. Peirce JM, Petry NM, Stitzer ML, et al. Effects of lower-cost incentives on stimulant abstinence in methadone maintenance treatment: a National Drug Abuse Treatment Clinical Trials Network study. *Arch Gen Psychiatry*. 2006;63(2):201-208. doi:10.1001/archpsyc.63.2.201

35. Poling J, Oliveto A, Petry N, et al. Six-month trial of bupropion with contingency management for cocaine dependence in a methadone-maintained population. *Arch Gen Psychiatry*. 2006;63(2):219-228. doi:10.1001/archpsyc.63.2.219

36. Knealing TW, Wong CJ, Diemer KN, Hampton J, Silverman K. A randomized controlled trial of the therapeutic workplace for community methadone patients: a partial failure to engage. *Exp Clin Psychopharmacol*. 2006;14(3):350-360. doi:10.1037/1064-1297.14.3.350

37. Gross A, Marsch LA, Badger GJ, Bickel WK. A comparison between low-magnitude voucher and buprenorphine medication contingencies in promoting abstinence from opioids and cocaine. *Exp Clin Psychopharmacol*. 2006;14(2):148-156. doi:10.1037/1064-1297.14.2.148

38. Brooner RK, Kidorf MS, King VL, Stoller KB, Neufeld KJ, Kolodner K. Comparing adaptive stepped care and monetary-based voucher interventions for opioid dependence. *Drug Alcohol Depend*. 2007;88 Suppl 2(Suppl 2):S14-S23. doi:10.1016/j.drugalcdep.2006.12.006

© 2021 Bolívar HA et al. *JAMA Psychiatry*.

39. Bickel WK, Marsch LA, Buchhalter AR, Badger GJ. Computerized behavior therapy for opioid-dependent outpatients: a randomized controlled trial. *Exp Clin Psychopharmacol*. 2008;16(2):132-143. doi:10.1037/1064-1297.16.2.132

40. Epstein DH, Schmittner J, Umbricht A, Schroeder JR, Moolchan ET, Preston KL. Promoting abstinence from cocaine and heroin with a methadone dose increase and a novel contingency. *Drug Alcohol Depend*. 2009;101(1-2):92-100. doi:10.1016/j.drugalcdep.2008.11.006

41. Chopra MP, Landes RD, Gatchalian KM, et al. Buprenorphine medication versus voucher contingencies in promoting abstinence from opioids and cocaine. *Exp Clin Psychopharmacol*. 2009;17(4):226-236. doi:10.1037/a0016597

42. Tuten M, Svikis DS, Keyser-Marcus L, O'Grady KE, Jones HE. Lessons learned from a randomized trial of fixed and escalating contingency management schedules in opioid-dependent pregnant women. *Am J Drug Alcohol Abuse*. 2012;38(4):286-292. doi:10.3109/00952990.2011.643977

43. Petry NM, Alessi SM, Ledgerwood DM. A randomized trial of contingency management delivered by community therapists. *J Consult Clin Psychol*. 2012;80(2):286-298. doi:10.1037/a0026826

44. Holtyn AF, Koffarnus MN, DeFulio A, et al. The therapeutic workplace to promote treatment engagement and drug abstinence in out-of-treatment injection drug

© 2021 Bolívar HA et al. *JAMA Psychiatry*.

users: a randomized controlled trial. *Prev Med*. 2014;68:62-70. doi:10.1016/j.ypmed.2014.02.021

45. Petry NM, Alessi SM, Barry D, Carroll KM. Standard magnitude prize reinforcers can be as efficacious as larger magnitude reinforcers in cocaine-dependent methadone patients. *J Consult Clin Psychol*. 2015;83(3):464-472. doi:10.1037/a0037888
46. McCaul ME, Stitzer ML, Bigelow GE, Liebson IA. Contingency management interventions: effects on treatment outcome during methadone detoxification. *J Appl Behav Anal*. 1984;17(1):35-43. doi:10.1901/jaba.1984.17-35
47. Silverman K, Wong CJ, Higgins ST, et al. Increasing opiate abstinence through voucher-based reinforcement therapy. *Drug Alcohol Depend*. 1996;41(2):157-165. doi:10.1016/0376-8716(96)01246-x
48. Preston KL, Umbricht A, Epstein DH. Methadone dose increase and abstinence reinforcement for treatment of continued heroin use during methadone maintenance. *Arch Gen Psychiatry*. 2000;57(4):395-404. doi:10.1001/archpsyc.57.4.395
49. Robles E, Stitzer ML, Strain EC, Bigelow GE, Silverman K. Voucher-based reinforcement of opiate abstinence during methadone detoxification. *Drug Alcohol Depend*. 2002;65(2):179-189. doi:10.1016/s0376-8716(01)00160-0

© 2021 Bolívar HA et al. *JAMA Psychiatry*.

50. Correia CJ, Dallery J, Katz EC, Silverman K, Bigelow G, Stitzer ML. Single- versus dual-drug target: effects in a brief abstinence incentive procedure. *Exp Clin Psychopharmacol*. 2003;11(4):302-308. doi:10.1037/1064-1297.11.4.302
51. Hser YI, Li J, Jiang H, et al. Effects of a randomized contingency management intervention on opiate abstinence and retention in methadone maintenance treatment in China. *Addiction*. 2011;106(10):1801-1809. doi:10.1111/j.1360-0443.2011.03490.x
52. Jiang H, Du J, Wu F, et al. Efficacy of contingency management in improving retention and compliance to methadone maintenance treatment: a random controlled study. *Shanghai Arch Psychiatry*. 2012;24(1):11-19. doi:10.3969/j.issn.1002-0829.2012.01.002
53. Chen W, Hong Y, Zou X, McLaughlin MM, Xia Y, Ling L. Effectiveness of prize-based contingency management in a methadone maintenance program in China. *Drug Alcohol Depend*. 2013;133(1):270-274. doi:10.1016/j.drugalcdep.2013.05.028
54. Ling W, Hillhouse M, Ang A, Jenkins J, Fahey J. Comparison of behavioral treatment conditions in buprenorphine maintenance. *Addiction*. 2013;108(10):1788-1798. doi:10.1111/add.12266

© 2021 Bolívar HA et al. *JAMA Psychiatry*.

55. Wang L, Wei X, Wang X, Li J, Li H, Jia W. Long-term effects of methadone maintenance treatment with different psychosocial intervention models. *PLoS One*. 2014;9(2):e87931. Published 2014 Feb 3. doi:10.1371/journal.pone.0087931
56. Jarvis BP, Holtyn AF, DeFulio A, et al. The effects of extended-release injectable naltrexone and incentives for opiate abstinence in heroin-dependent adults in a model therapeutic workplace: A randomized trial. *Drug Alcohol Depend*. 2019;197:220-227. doi:10.1016/j.drugalcdep.2018.12.026
57. Shoptaw S, Rotheram-Fuller E, Yang X, et al. Smoking cessation in methadone maintenance. *Addiction*. 2002;97(10):1317-1325. doi:10.1046/j.1360-0443.2002.00221.x
58. Dunn KE, Sigmon SC, Thomas CS, Heil SH, Higgins ST. Voucher-based contingent reinforcement of smoking abstinence among methadone-maintained patients: a pilot study. *J Appl Behav Anal*. 2008;41(4):527-538. doi:10.1901/jaba.2008.41-527
59. Dunn KE, Sigmon SC, Reimann EF, Badger GJ, Heil SH, Higgins ST. A contingency-management intervention to promote initial smoking cessation among opioid-maintained patients. *Exp Clin Psychopharmacol*. 2010;18(1):37-50. doi:10.1037/a0018649
60. Tuten M, Fitzsimons H, Chisolm MS, Nuzzo PA, Jones HE. Contingent incentives reduce cigarette smoking among pregnant, methadone-maintained women:

© 2021 Bolívar HA et al. *JAMA Psychiatry*.

results of an initial feasibility and efficacy randomized clinical trial. *Addiction*. 2012;107(10):1868-1877. doi:10.1111/j.1360-0443.2012.03923.x

61. Sigmon SC, Miller ME, Meyer AC, et al. Financial incentives to promote extended smoking abstinence in opioid-maintained patients: A randomized trial. *Addiction*. 2016;111(5):903-912. doi:10.1111/add.13264
62. Jones HE, Haug NA, Stitzer ML, Svikis DS. Improving treatment outcomes for pregnant drug-dependent women using low-magnitude voucher incentives. *Addict Behav*. 2000;25(2):263-267. doi:10.1016/s0306-4603(98)00119-1
63. Rhodes GL, Saules KK, Helmus TC, et al. Improving on-time counseling attendance in a methadone treatment program: a contingency management approach. *Am J Drug Alcohol Abuse*. 2003;29(4):759-773. doi:10.1081/ada-120026259
64. Kidorf M, Brooner RK, Gandotra N, et al. Reinforcing integrated psychiatric service attendance in an opioid-agonist program: a randomized and controlled trial. *Drug Alcohol Depend*. 2013;133(1):30-36. doi:10.1016/j.drugalcdep.2013.06.005
65. Kidorf M, Brooner RK, Leoutsakos JM, Peirce J. Treatment initiation strategies for syringe exchange referrals to methadone maintenance: A randomized clinical trial. *Drug Alcohol Depend*. 2018;187:343-350. doi:10.1016/j.drugalcdep.2018.03.009

© 2021 Bolívar HA et al. *JAMA Psychiatry*.

66. Preston KL, Silverman K, Umbricht A, DeJesus A, Montoya ID, Schuster CR. Improvement in naltrexone treatment compliance with contingency management. *Drug Alcohol Depend*. 1999;54(2):127-135. doi:10.1016/s0376-8716(98)00152-5

67. Sorensen JL, Haug NA, Delucchi KL, et al. Voucher reinforcement improves medication adherence in HIV-positive methadone patients: a randomized trial. *Drug Alcohol Depend*. 2007;88(1):54-63. doi:10.1016/j.drugalcdep.2006.09.019

68. Everly JJ, DeFulio A, Koffarnus MN, et al. Employment-based reinforcement of adherence to depot naltrexone in unemployed opioid-dependent adults: a randomized controlled trial. *Addiction*. 2011;106(7):1309-1318. doi:10.1111/j.1360-0443.2011.03400.x

69. DeFulio A, Everly JJ, Leoutsakos JM, et al. Employment-based reinforcement of adherence to an FDA approved extended release formulation of naltrexone in opioid-dependent adults: a randomized controlled trial. *Drug Alcohol Depend*. 2012;120(1-3):48-54. doi:10.1016/j.drugalcdep.2011.06.023

70. Dunn KE, Defulio A, Everly JJ, et al. Employment-based reinforcement of adherence to oral naltrexone treatment in unemployed injection drug users. *Exp Clin Psychopharmacol*. 2013;21(1):74-83. doi:10.1037/a0030743

© 2021 Bolívar HA et al. *JAMA Psychiatry*.

71. Weaver T, Metrebian N, Hellier J, et al. Use of contingency management incentives to improve completion of hepatitis B vaccination in people undergoing treatment for heroin dependence: a cluster randomised trial. *Lancet*. 2014;384(9938):153-163. doi:10.1016/S0140-6736(14)60196-3
72. DeFulio A, Silverman K. Employment-based abstinence reinforcement as a maintenance intervention for the treatment of cocaine dependence: post-intervention outcomes. *Addiction*. 2011;106(5):960-967. doi:10.1111/j.1360-0443.2011.03364.x
73. Kosten T, Poling J, Oliveto A. Effects of reducing contingency management values on heroin and cocaine use for buprenorphine- and desipramine-treated patients. *Addiction*. 2003;98(5):665-671. doi:10.1046/j.1360-0443.2003.00380.x
74. Dunn K, DeFulio A, Everly JJ, et al. Employment-based reinforcement of adherence to oral naltrexone in unemployed injection drug users: 12-month outcomes. *Psychol Addict Behav*. 2015;29(2):270-276. doi:10.1037/adb0000010

© 2021 Bolívar HA et al. *JAMA Psychiatry*.
